# Affinity-based controlled release of interleukin-4 from scaffolds via biotin-streptavidin interactions for immunomodulation

**DOI:** 10.1016/j.jconrel.2025.113943

**Published:** 2025-06-09

**Authors:** Victoria A. Nash, Juan F. Cortes-Troncoso, Phoebe E. Chua, Kara L. Spiller

**Affiliations:** School of Biomedical Engineering, Sciences and Health Systems, Drexel University, Philadelphia, PA 19104, United States of America

**Keywords:** Drug delivery, Immunomodulation, Macrophage, Controlled release, Biotin avidin

## Abstract

Immunomodulatory cytokines like interleukin-4 (IL-4) can modulate host immune cell behavior to improve tissue integration, but versatile strategies for modifying complex biomaterials to control the release of such cytokines are limited. Bioconjugation strategies using biotin-avidin interactions offer a promising approach since biotin can be conjugated to proteins, biomaterials, and even cells, without compromising their function. Although it is known that conjugation of biotin to large biomolecules reduces its binding affinity for avidin and avidin variants, the potential to control the release of biotinylated molecules by leveraging these changes in affinity interactions has not been thoroughly investigated. Moreover, the effects of biotin and avidin variants on innate immunity are poorly understood. Therefore, the goals of this study were to determine if biotin-avidin interactions could be manipulated to control the release of IL-4 from biomaterials and to investigate the subsequent effects on primary human macrophage phenotype. First, we characterized the effects of soluble biotin, avidin, and avidin variants, streptavidin and CaptAvidin, on the phenotype of primary human macrophages from 8 different donors using RNA sequencing, finding that CaptAvidin influenced macrophage gene expression much more than the other variants. Then, after evaluating how bioconjugation parameters influenced biotin density and avidin variant binding to porous gelatin scaffolds, we found that biotin-avidin affinity interactions sustained the release of biotinylated IL-4 (bIL-4) from biotinylated and desthiobiotinylated scaffolds bound with either avidin or streptavidin for up to 14 days in vitro. Finally, we measured the response of primary human macrophages from 5 donors to the bIL-4-releasing scaffolds, finding an increase in reparative macrophage phenotype gene expression when bIL-4 was released via biotin-streptavidin interactions compared to scaffolds that relied solely on desorption-based release. These results highlight how biotin-streptavidin interactions can be leveraged for controlled release to achieve an immunomodulatory drug delivery system.

## 1. Introduction

Macrophages are critical regulators of all other cells throughout every stage of the healing process, including the response to implanted biomaterials (reviewed in [1,2]). In the initial response to injury, tissue resident macrophages secrete inflammatory cytokines and chemokines to recruit circulating immune cells to the site of injury, which differentiate into macrophages that overwhelmingly replace tissue resident macrophages in large numbers (reviewed in [3]). Monocyte-derived macrophages then polarize to a myriad of different phenotypes, depending upon local cues [4,5]. Numerous studies have shown that macrophages must transition from pro-inflammatory, which are critical for the initiation of wound healing processes [4,6], to a diverse population of reparative phenotypes that are required for resolution of inflammation and successful wound healing ([7,8] and reviewed in [9]). Biomaterials are often used to provide scaffolding to facilitate tissue repair in very large injuries. The response of macrophages to implanted biomaterials is a major determinant of biomaterial success or failure, with biomaterials that fail to support a switch from pro-inflammatory to reparative macrophages resulting in chronic inflammation, fibrosis, and impaired tissue repair (reviewed in [10,11]). Therefore, an emerging field of research is the design of biomaterials that control macrophage phenotype, to either sequentially promote pro-inflammatory and reparative phenotypes ([12,13] and reviewed in [14]) or to directly promote reparative phenotypes ([15,16] and reviewed in [17]).

Although there are several different types of reparative macrophage phenotypes, one that is particularly important in wound healing and vascularization is stimulated by interleukin-4 (IL-4) [18,19]. IL-4-stimulated macrophages secrete factors involved in stabilization and growth of blood vessels [8] and they cause endothelial cells [4] and fibroblasts [20] to take on maturation-associated behaviors in vitro. We and others have shown that sustained release of IL-4 from biomaterials can reduce the foreign body response and/or promote tissue vascularization in vivo [20–23]. Approaches used to control the release of IL-4 and other immunomodulatory cytokines and drugs from biomaterials include hydrogels, surface coatings, or encapsulating cytokines directly within a biodegradable component [13,24–26]. However, these approaches are not amenable to a variety of biomaterials. There is a need for a platform that can be easily applied to different biomaterials post manufacturing to deliver cytokines like IL-4 for better biomaterial integration and vascularization.

Affinity-based controlled release systems, such as those involving heparin or albumin, have been investigated as an approach for immunomodulatory drug delivery from biomaterials [27,28]. However, there is limited control over the release profiles from these systems, and many therapeutic drugs and proteins do not have strong affinity for heparin or albumin. Custom-designed peptides and aptamers have been devised to enable affinity-based release systems [29–31], but these systems can be challenging to apply to complex biomaterials. Biotin-avidin affinity is a favorable option because biotin can be directly conjugated to proteins [12], biomaterials [32,33], and even cells [34,35] without altering their bioactivity, which allows highly specific and strong binding to avidin and avidin variants like streptavidin. Avidin and streptavidin have extremely high affinity for biotin (K_D_ = 10^−15^ M for avidin and K_D_ = 10^–14 to−15^ for streptavidin) [36,37], and they have 4 binding sites for biotin, allowing biotinylated drugs and proteins to be attached to biotinylated biomaterials via avidin or avidin variants. In addition to wild type avidin and streptavidin, others have genetically or chemically modified these variants to not only suit their particular application, but to also decrease binding capacity (reviewed in [38]). One such example is commercially available CaptAvidin, a nitrated version of avidin that has been reported to bind only 2.7 mols of biotin compared to the 4 mols of biotin that wild type avidin can theoretically bind [39]. Additionally, CaptAvidin has an affinity for biotin similar to wild type avidin at low and neutral pH, but drastically decreased affinity at high pH (K_D_ = 10^−9^ M at >pH 9). Furthermore, this modified avidin variant demonstrates an increase in dissociation events in the presence of free biotin compared to wild type avidin, even at low and neutral pH [40], further indicating a decrease in affinity for biotin compared to avidin. Another way to manipulate the biotin-avidin affinity interaction is to use the precursor to biotin, desthiobiotin, because of its lower affinity to avidin and its variants (K_D_ = 10^−13^ M) [41]. These modifications that influence affinity interactions between biotin and avidin variants suggest potential for leveraging them to control the release of proteins and drugs from biomaterials, but this strategy has not been thoroughly investigated. Furthermore, while biotin-avidin interactions are so strong they are often considered essentially irreversible, some studies have shown that when biotin is conjugated to larger molecules, its binding to affinity-modified avidins decreases [42,43]. Others have leveraged this reduction in affinity of biotinylated molecules to release from biomaterials via the introduction of free biotin into the system, which effectively displaces the lower affinity biotin conjugate from avidin [42,44]. Previously, we have used biotin-streptavidin and biotin-CaptAvidin interactions to add IL-4 to a porous gelatin scaffold and demonstrated proof of concept that this strategy has the potential to control macrophage phenotype [45]. However, controlled release of biotinylated molecules from a biomaterial using affinity for avidin variants in the absence of free biotin has not yet been demonstrated, and detailed characterization of the response of macrophages to the system has not been conducted.

Finally, since macrophages can respond to multiple environmental cues at once, it is possible that avidin and its variants could influence macrophage phenotype independently of any additional cytokines. Previously, avidin, derived from chicken egg white, was used as a model adjuvant [46] and later as a protein carrier for novel adjuvants [47] for vaccines. It was ultimately phased out of vaccine design because the avidin complexes did not induce immune memory [48]. Variants of avidin are used in many different applications (reviewed in [49]), but the immunomodulatory properties of avidin and these variants have only been investigated in the context of adaptive immunity [50,51], not innate immunity. Since anti-avidin antibodies can be raised in response to exposure to avidin in animals and humans [46,52], it is possible that avidin and its variants could influence macrophage phenotype, but it has not yet been explored. Therefore, there is a need to investigate how avidin and its variants affect macrophage phenotype to thoughtfully select a variant for biomaterial modification.

**Fig. 1.**
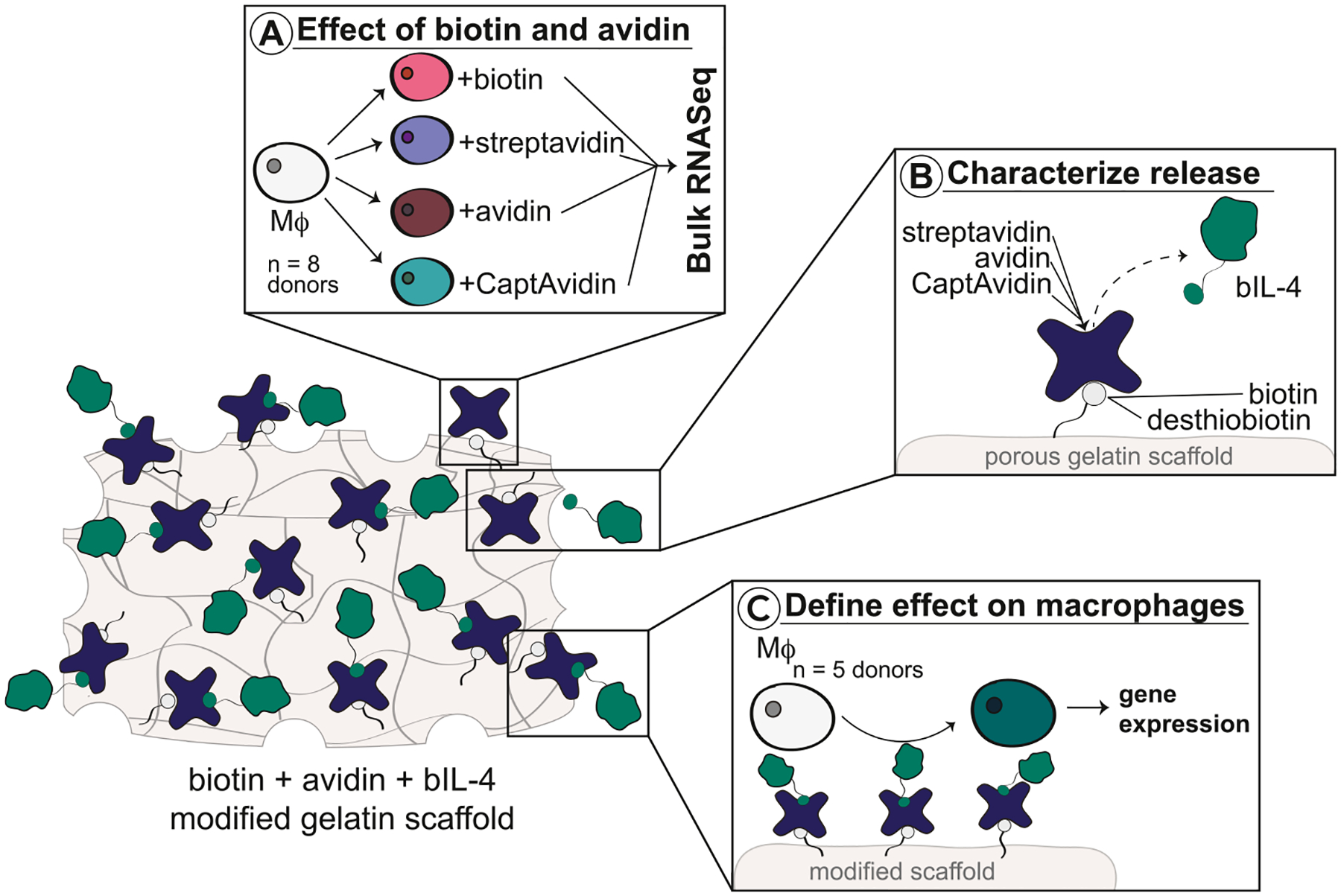
Experimental design. The goals of this study were to (A) Define effect of biotin, avidin, streptavidin, and CaptAvidin on unactivated macrophages using bulk RNASequencing; (B) Characterize material properties and subsequent spontaneous release of biotinylated IL-4 from modified scaffolds; (C) Investigate presentation of bIL-4 by biotin-streptavidin modified porous gelatin scaffolds on macrophages using quantitative real-time PCR. Mϕ, macrophage. bIL-4, biotinylated IL-4.

In this study, we investigated how bioconjugation parameters influence the binding of avidin variants and the release of biotinylated IL-4 (bIL-4) from biotinylated porous gelatin scaffolds, selected as model biomaterials, and the effect this platform has on the phenotype of primary human macrophages using multiple human donors (Fig. 1). We hypothesized that biotin-avidin affinity interactions would release bIL-4 more slowly than desorption-based release and promote the reparative macrophage phenotype. First, we investigated the effects of soluble avidin, streptavidin, CaptAvidin, and two doses of biotin on macrophage phenotype using bulk RNA sequencing (RNASeq) (Fig. 1A). Next, we thoroughly investigated how the type of biotin variant (biotin or desthiobiotin) conjugated to scaffolds, the type of avidin variant (avidin, streptavidin, and CaptAvidin), and the dose of b-IL4 influenced the release of bIL-4 (Fig. 1B) and the resulting effects on primary human macrophage phenotype (Fig. 1C).

## 2. Methods

### 2.1. Monocyte isolation and macrophage differentiation

Peripheral blood-derived monocytes (PBMCs) were either purchased directly from the University of Pennsylvania Human Immunology Core (donor information in Suppl. Table S1, 8 donors, for RNAseq studies) or isolated from buffy coats purchased from the New York Blood Center (Suppl. Table S2, 5 donors, for scaffold studies) according to our previously described methods [53] and then frozen for future use. To freeze PBMCs, cells were counted, pelleted, and resuspended at 10 × 10^7^ cells / mL in freezing media, made up of 10 % dimethyl sulfoxide (DMSO, Sigma) and fetal bovine serum (FBS, Gibco). PBMCs were brought down to −80 °C overnight, then moved to liquid nitrogen storage. To thaw cells in preparation for monocyte isolation, a vial of frozen PBMCs was submerged in a water bath at 37 °C until almost completely thawed. Cells were then aliquoted into 1 mL of fetal bovine serum (FBS) warmed to 37 °C and additional 1 mL of warmed FBS was used to wash the freezing vial and added to the cell suspension. Following that, prewarmed isolation media made up of RPMI 1640, 10 % FBS, and 1 % penicillin/streptomycin (P/S) was added dropwise to the cell suspension to reach a final volume of 30 mL. Cells were then centrifuged at 300 ×*g* (Eppendorf 5910 Ri) for 10 min at 4 °C to remove any remaining DMSO. After resuspending in isolation media, cells were counted and centrifuged again for 5 min at 485 ×*g* and 4 °C for an additional wash step. During the final wash step, monocyte isolation columns and kits were set up according to manufacturer’s protocols (using a negative selection Pan Monocyte Isolation Kit from Miltenyi Biotec). Isolated monocytes were spun down at 485 ×*g* (Eppendorf 5910 Ri) for 10 min at 4 °C then resuspended in prewarmed culture media made up of RPMI 1640, 10 % human serum (HS) and 1 % P/S. Cells were counted and diluted to 1 × 10^6^ cells/mL and cultured as previously described at 1 × 10^6^ cells/mL on non-tissue-culture treated polystyrene plates (Corning) [8]. Monocytes were differentiated into unactivated macrophages by adding 20 ng/mL of macrophage colony-stimulating factor (M-CSF, Peprotech) for up to 7 days, with media changes on days 3 and 5. All cells adhered to the non-tissue culture-treated plastic, indicating a pure population of mature macrophages.

### 2.2. Stimulating macrophages with soluble biotin and avidin variants

To stimulate human macrophages with avidin variants, doses of biotin and avidin variants were calculated. Two doses of biotin were investigated. The low dose of biotin was equivalent to the biotin density previously reported to be conjugated to porous gelatin scaffolds (5 mm diameter × 8 mm height) where a 1-fold-molar-excess (FME) of Sulfo-NHS-LC-biotin (Pierce Premium Grade, Thermo Fisher) biotinylation reagent was used (3.37 × 10^−7^ M) [45]. To investigate the effect of biotin at a higher dose, a 10× higher dose was used (3.37 × 10^−6^ M). The dose of avidin variants was calculated assuming a 1:1 M ratio of binding and using the lower biotin dose [45]. Doses that corresponded to 3.37 × 10^−7^ M per scaffold were 0.66 μg/mL for avidin (ThermoFisher) and CaptAvidin (invitrogen) and 0.53 μg/mL for streptavidin (Molecular Probes) (MW = 55,000 g/mol) [37].

Monocytes from 8 donors (4 male and 4 female) were seeded at 1 × 10^6^ cells/mL in 500 μL of media (RPMI +10 % HS + 1 % P/S + M-CSF) and allowed to differentiate into macrophages for 6 days with a media change at day 3. Soluble biotin or avidin variants were then added directly to the media and cells were cultured for and additional 24 h. Macrophages were then collected by gentle scraping and centrifuged (Sorvall Legend Micro 17R) at 200 ×g for 5 min. Supernatant was removed and the cell pellet was vortexed quickly before and after adding lysis buffer (RNAqueous Micro Total RNA Isolation Kit, ThermoFisher). Cell lysates were stored at −80 °C until RNA extraction.

### 2.3. RNA extraction, library construction, quality control, and bulk RNASeq analysis

After cell lysates were thawed and quickly vortexed, total RNA was extracted using the RNAqueous Micro Kit (invitrogen) according to the manufacturer’s protocol but heating the Elution Solution to 65 °C. RNA was quantified using the Take3 plate from BioTek and considered pure if the 260/280 ratio was ~2. Samples were then stored at −80 °C for Bulk RNA Sequencing (RNASeq). Total RNA was sent to SingulOmics Corporation (Bronx, NY) for RNASeq. Briefly, and following previously published methods [54], messenger RNA was extracted using poly-T oligo-attached magnetic beads, then fragmented. Complementary DNA (cDNA) was quantified using real-time polymerase chain reaction and fragment size was measured using a Bioanalyzer. The cDNA library was then constructed and analyzed with Qubit. Quantified libraries were then pooled and sequenced on Illumina platforms, based on sequencing by synthesis and targeting 6 million read pairs per sample.

Data were transformed to raw reads by CASACA base recognition and the error rate for each base was calculated according to Eq. 1, where *e* represents the sequencing error rate. Guanine-cytosine base pair content was also assessed to ensure quality reads.


(1) $$\text{Qphred}=-{10\text{log}}_{10}e$$

Sequencing reads were then filtered to remove low-quality reads by first removing reads with adapter contamination, then reads where uncertain nucleotides made up more than 10 % of the read and finally reads where low-quality nucleotides (base quality <5 %) were responsible for greater than 50 % of the read. Alignments were then performed using HISAT2 v2.0.5 to the reference human genome [55]. To count read numbers mapped to a gene, featureCounts v1.5.0-p3 was used and fragments per kilobase of transcript sequence per million base pairs sequenced (FPKM) was calculated based on the length of the gene and read counts mapped to it. Differential expression analysis was then conducted using DESeq2 R package (version 1.20.0) and *p*-values were adjusted using the Benjamini and Hochberg procedure for false discovery rate, where genes with an adjusted *p*-value less than or equal to 0.05 were considered differentially expressed. Outliers were identified using the robust regression and outlier removal (ROUT) method with q = 1 %. Pathway analyses were assessed using the clusterProfiler R package and considered significantly enriched if the corrected p-value was less than 0.05. Gene set enrichment analysis (GSEA) using each pathway analysis was run separately using the analysis tool provided by UC San Diego and the Broad Institute [54,56]. Data have been deposited in the Gene Expression Omnibus (GEO).

### 2.4. Biotinylation of scaffolds

Porous gelatin scaffolds were biopsy-punched from a sheet (2 mm thick, Surgifoam, Ethicon) to 5 mm diameter and crosslinked in a solution of 1.15 mg/mL 1-ethyl-3(3-dimethylaminopropyl) carbodiimide (EDC) and 0.261 mg/mL N-hydroxysuccinimide (NHS) in PBS at room temperature (RT) for 2 h rotating at 25 rpm (RPM) on a tube rotator as previously described [57]. Scaffolds were then washed in PBS 5 times, 5 min per wash at RT and 25 RPM on a tube rotator to remove any unreacted reagents and stored at 4 °C until further use. Scaffolds used in bioactivity studies were created using sterile filtered EDC and NHS solutions and sterile products in a biosafety cabinet using aseptic technique. Scaffolds were incubated for 1 h at RT at 25 RPM on a tube rotator with 0.1-, 1-, or 10-fold-molar-excess (FME) of Sulfo-NHS-LC-Biotin (Pierce Premium Grade, ThermoFisher) or EZ-Link^™^ Sulfo-NHS-LC-Desthiobiotin (No Weigh Format, ThermoFisher) for covalent conjugation to available primary amines on the scaffolds as previously described [45]. FME was determined as mols of biotinylation reagent to approximate mols of gelatin in the scaffold, estimated as 1.8 × 10^−7^ mol per mm^3^ of scaffold. After 1-h, biotinylated scaffolds were washed 5 times, 5 min per wash at RT and 25 RPM on a tube rotator, in PBS to remove any unbound biotinylation reagent. For bioactivity studies, biotinylation was carried out using sterile reagents in a biosafety cabinet using aseptic techniques.

### 2.5. Binding avidin variants to biotinylated scaffolds

Biotinylated or desthiobiotinylated scaffolds were incubated with avidin, streptavidin or CaptAvidin at a concentration of 0.625 mg/mL at RT for 1 h and 25 RPM on a tube rotator. To prepare controls in which biotinylated cytokine would be passively adsorbed to the scaffolds, scaffolds were treated the same but with no avidin variant. Scaffolds were then washed 5 times, 5 min per wash at RT and 25 RPM, in PBS. For bioactivity studies, these steps were carried out under sterile conditions using sterile avidin variants and sterile PBS. To confirm binding of avidin variants to biotinylated scaffolds, fluorescent avidin variants at the same concentrations were used. For CaptAvidin modified scaffolds, CaptAvidin was first conjugated with primary amine reactive fluorescent Alexa Fluor 488 (Alexa Fluor^™^ 488 NHS Ester (Succinimidyl Ester, ThermoFisher) by following the manufacturer’s protocol. Briefly, CaptAvidin was incubated with the reactive Alexa Fluor at 5 mg/mL for 1 h at RT and 25 RPM on a tube rotator. Following the incubation, labeled CaptAvidin was dialyzed (7000 Da molecular weight cutoff dialysis cassette, ThermoFisher) for 4 h at room temperature in 500 times excess PBS, with a media change every 2 h, before dialyzing overnight to remove any unconjugated reactive Alexa Fluor. Following dialysis, fluorescent CaptAvidin was incubated with scaffolds for 1 h at RT and 25 RPM on a tube rotator, followed by 5 washes, 5 min per wash and 25 RPM, with PBS. Commercially available fluorescent avidin (Alexa Fluor 488 conjugate, invitrogen) and fluorescent streptavidin (Alexa Fluor 488 conjugate, invitrogen) were purchased and added to biotinylated scaffolds in the same manner as the fluorescent CaptAvidin. After the final wash step, fluorescently labeled avidin-modified scaffolds were added to polystyrene assay plates (Corning) and read in a plate reader (BioTek Gen5) at 485 nm excitation and 520 nm emission for fluorescent avidin and streptavidin and 490 nm excitation and 525 nm emission for fluorescent CaptAvidin.

### 2.6. Quantifying release of bIL-4 from biotin-avidin modified scaffolds

Immediately following binding with avidin variants, commercially available biotinylated IL-4 (bIL-4) (ACROBiosystems) was added to each scaffold by incubating the scaffolds in bIL-4 solution for 1 h at RT and 25 RPM. Low dose scaffolds were incubated with 1.14 × 10^−7^ M (2,000 ng/mL) of bIL-4 in 200 μL of solution and high dose scaffolds were incubated with 5.68 × 10^−7^ M (10,000 ng/mL) of bIL-4200 μL of solution. Scaffolds were then washed 3 times, 5 min per wash, before immediately preceding to release studies or macrophage bioactivity studies.

Release studies were conducted in 200 μL of either 1× PBS or a 10 mM solution of free biotin in 1× PBS at 37 °C and shaking at 25 RPM. At each time point, the entire supernatant was removed and replaced with the appropriate buffer (either 1× PBS or 10 mM free biotin). The amount of bIL-4 in the supernatant was quantified using enzyme-linked immunosorbent assay (ELISA) according to the manufacturer’s instructions (Peprotech). All release studies were carried out for 14 days and bIL-4 was measured in all supernatants. If more than 60 % of replicates in a group at a given time point were below the limit of detection for the ELISA (15.6 pg/mL to 1000 pg/mL), then the group was considered to have reached the end of its release profile at the preceding time point. To calculate fractional release profiles, the mass released at each time point was divided by the total mass released.

### 2.7. Response of macrophages to bIL-4 modified scaffolds

Macrophages from 5 donors (Suppl. Table S2) were collected after 5 days of culture with M-CSF by following the manufacturer’s protocol using an enzymatic cell detachment solution (ACCUTASE, Miltenyi). Cells were then seeded onto modified scaffolds at 3 × 10^5^ cells per scaffold in 10 μL of culture media (RPMI 1640 + 10 % HS + 1 % Pen/Strep). Macrophages were allowed to adhere to scaffolds for 4 h at 37 °C and 5 % CO_2_ before an additional 300 μL of culture media with 20 ng/mL M-CSF (Peprotech) was added to fully submerge scaffolds. Media was fully exchanged on day 2. Cell-seeded scaffolds were collected on day 4 for quantitative reverse transcription PCR (qRT-PCR) analysis.

### 2.8. Quantitative reverse transcription polymerase chain reaction (qRT-PCR) analysis

RNA extraction from macrophages seeded onto modified scaffolds was carried out using the Bead Genie bead beater (Scientific Industries, USA) and RLT Plus Lysis Buffer (Qiagen, Germany). Briefly, scaffolds were placed into ZR BashingBead Lysis tube containing 2.0 mm size beads (Zymo Research, USA) and 600 μL of RLT Plus Lysis Buffer was added. Samples were agitated for 3 cycles of 1-min, with 30 s of rest between cycles. Samples were then incubated for 1 min on ice before centrifuging at RT for 1 min at 16,000 g (Sorvall Legend Micro 17R, ThermoScientific). The supernatant was carefully collected, and total RNA was purified from it. Total RNA purification was carried out using the RNAqueous Micro kit following manufacturer’s protocols (invitrogen, CA, USA). RNA purity was assessed using the 260/280 nm ratio as described above and RNA integrity (RIN) was measured by an Agilent 2100 Bioanalyzer using the Agilent RNA 6000 Nano Kit and following manufacturers recommendations (Agilent Technologies, Canada). RNA was considered high quality if the RIN value was above 8.0. Template cDNA was obtained by reverse transcription of 200 ng total RNA with High-Capacity cDNA Reverse Transcription Kit (Applied Biosystems). From the template cDNA created, 10 ng per reaction was used for the qRT-PCR reaction in triplicate using Fast SYBR Green Master Mix (invitrogen, CA, USA). All qRT-PCR reactions were performed on a qTOWER3 G real-time PCR thermal cycler (Analytik Jena). Expression of mRNA was determined relative to four housekeeping genes (*VPS29, SDHA, HPRT1*, and *UBE2D2*) using primers from ThermoFisher (Suppl. Table S3). The relative expression of genes was expressed in comparison to the average of 4 housekeeping genes using the 2^−ΔCt^ method.

### 2.9. Statistical analysis

RNASeq analysis was conducted with *n* = 8 replicates per group, one per human donor. Binding of avidin variants was analyzed with *n* = 10 replicates per group. Release of bIL-4 as a function of avidin variant was conducted with *n* = 5 replicates, and repeated as a function of bIL-4 dose with n = 10 replicates. Data were considered normal if they passed the D’Agostino-Pearson, Anderson-Darling, Shapiro-Wilk, and Kolmogorov-Smirnov tests. Release data at individual time points were analyzed using a way-way analysis of variance (ANOVA) with Tukey’s post hoc analysis. Expression of individual genes was analyzed using repeated measures (RM) ANOVA with the Geisser-Greenhouse correction, followed by Tukey’s post hoc test. Comparisons were considered significantly different if *p* < 0.05.

## 3. Results

### 3.1. CaptAvidin influences macrophage phenotype more than streptavidin and avidin

**Fig. 2.**
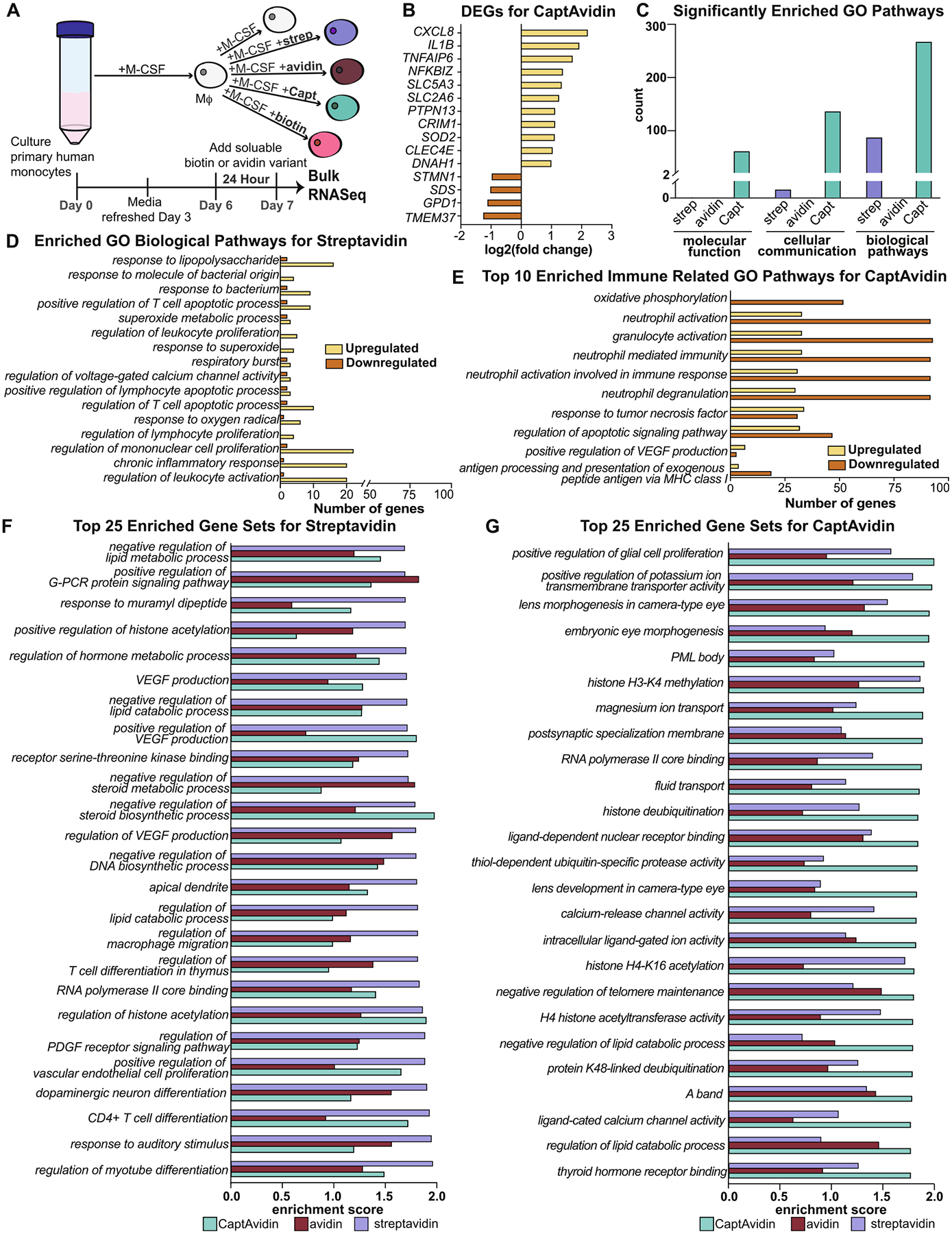
RNASeq analysis of biotin, avidin, streptavidin, and CaptAvidin on macrophages. (A) Experimental design. (B) Differentially expressed genes for CaptAvidin treatment compared to untreated macrophages that were protein coding, with a p-adjusted<0.05 and an absolute log2(fold change) > 1. (C) Number of significantly enriched gene ontology (GO) pathways for streptavidin, CaptAvidin, and avidin treatments. (D) Number of up- and down-regulated genes in significantly enriched GO biological pathways for macrophages treated with streptavidin. (E) Number of up- and down-regulated genes in the top 10 most significantly enriched immune related GO pathway for macrophages treated with CaptAvidin. (F) Comparison of top 25 most enriched gene sets from GSEA of GO pathways for streptavidin to treatment with avidin or CaptAvidin. (G) Comparison of top 25 most enriched gene sets from GSEA of GO pathways for CaptAvidin to treatment with avidin or streptavidin. *n* = 8 donors, 4 male and 4 female. Mϕ, macrophage. Strep, streptavidin. Capt, CaptAvidin. GO, gene ontology. GSEA, gene set enrichment analysis.

First, we set out to understand how the components of the biotin-avidin system influenced macrophage phenotype even without the addition of immunomodulatory cytokines. We added soluble avidin, streptavidin, CaptAvidin, and biotin (in two doses) to monocyte-derived macrophages from 8 different human donors, 4 male and 4 female with ages ranging from 25 to 55 (Suppl. Table S1). After stimulating macrophages with biotin or avidin variant for 24 h, we analyzed gene expression by RNASeq one day later (Fig. 2A). There were 175 differentially expressed genes (DEGs) for CaptAvidin treatment compared to the untreated control with an adjusted *p*-value<0.05 (Suppl. Table S4) but no DEGs for streptavidin or avidin. Within the DEGs for CaptAvidin, we looked at protein-coding DEGs with a log2(fold change) > 1 and found 15 genes, 11 that were upregulated and 4 that downregulated, compared to untreated macrophages (Fig. 2B). Of these, *CXCL8, IL1B, NFKBIZ*, *TNFAIP6*, and *CRIM1* are generally associated with inflammation, and these genes were significantly upregulated in the CaptAvidin treated group compared to untreated control macrophages, but only *IL1B* was expressed at significantly higher levels compared to streptavidin and avidin treated macrophages (Suppl. Fig. S1). Additionally, in at least 6 of the 8 donors, when using gene set enrichment analysis (GSEA) to determine which genes significantly influenced the top 50 enriched GO pathways for macrophages treated with CaptAvidin, those same genes were the most highly expressed (Suppl. Fig. S2). Other genes upregulated by CaptAvidin were tied to various cellular activities, while the 4 downregulated genes were generally associated with metabolism (Suppl. Fig. S1).

Gene ontology (GO) analysis also showed that CaptAvidin had the strongest effect on macrophage gene expression (Fig. 2C). Treatment with CaptAvidin compared to the untreated control resulted in over 400 significantly enriched GO pathways, while treatment with streptavidin influenced just over 100 pathways, and no pathways were significantly enriched for avidin treated macrophages. Further analysis of the biological pathways that were significantly enriched revealed that streptavidin treatment generally upregulated pathways associated with inflammation related pathways (Fig. 2D) while CaptAvidin treatment generally downregulated pathways associated with innate immunity (Fig. 2E). Among the 25 most enriched gene sets from the GO pathway analysis of streptavidin-treated macrophages, gene sets associated with both the innate and adaptive immune systems were significantly influenced, while gene sets enriched by CaptAvidin treatment were less obviously linked to immune cell function (Fig. 2F and G).

Other pathway analyses further highlighted the influence of CaptAvidin on macrophages. CaptAvidin was the only variant with enriched pathways found in Kyoto Encyclopedia of Genes and Genomes (KEGG), Disease Ontology (DO), and DisGeNET analyses. In KEGG enrichment analysis, pathways ranging from apoptosis to tuberculosis were down-regulated with CaptAvidin treatment (Suppl. Fig. S3 and Suppl. Tables S5, S6, S7). While CaptAvidin treatment impacted over 100 different pathways in Reactome Enrichment Analysis (Suppl. Table S8), avidin treatment impacted only 3 pathways (*elastic fiber formation, interferon gamma signaling*, and *TGF-beta receptor signaling in epithelial to mesenchymal transition*). Streptavidin did not significantly enrich any of these pathways. Taken together, multiple pathway analyses suggest that stimulation with CaptAvidin had stronger effects on macrophage gene expression compared to either avidin or streptavidin, although no treatment was completely without effects.

To understand the effect of biotin on macrophages, we interrogated two different doses: a dose correlating to the mass of biotin on a porous scaffold determined in previous work [45] (low dose) and a dose 10× greater (high dose), although those scaffolds were thicker than those used in the present study, so the biotin concentration would be higher. There was no effect of the low dose of biotin on macrophages, but there were some significant effects at the high dose. The 30 significantly enriched GO pathways observed at this high dose of biotin were generally associated with biological processes and cellular communication (Suppl. Fig. S4). Closer analysis of the gene set enrichment analysis (GSEA) of the top 50 significantly enriched GO pathways showed that the most significantly enriched genes in most pathways were non-coding (Suppl. Fig. S5). Focusing on the gene level, both doses of biotin did not result in any DEGs after applying a false discovery calculation using the Benjamini-Hochberg procedure. Overall, these results show that biotin has minimal effects on macrophage phenotype, at least in terms of gene expression at 24 h, even at a relatively high dose.

### 3.2. bIL-4 is spontaneously released from biotinylated scaffolds via biotin-avidin affinity interactions

**Fig. 3.**
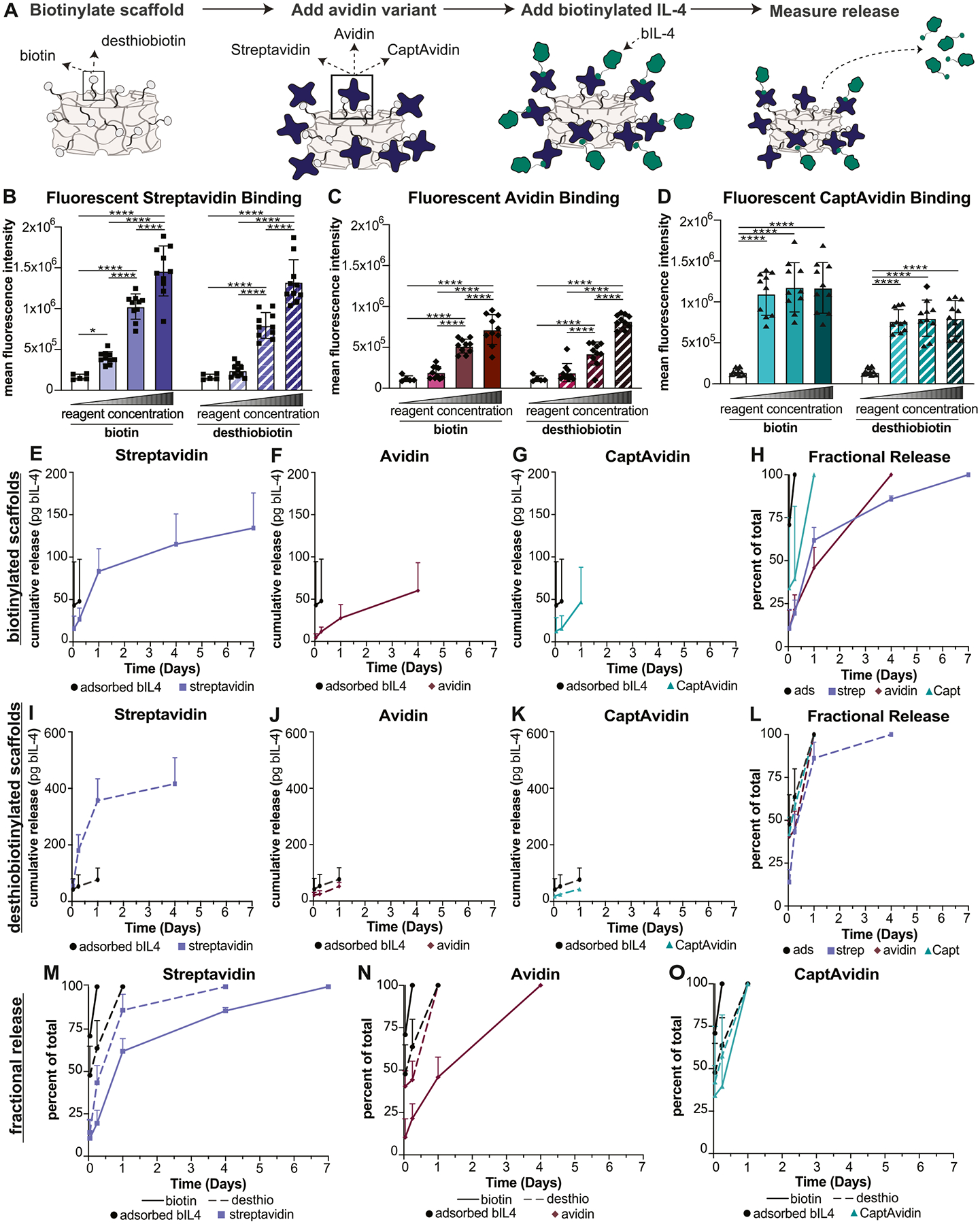
Characterization of bIL-4-releasing porous gelatin scaffolds. (A) Experimental design and variables investigated. (B–D) Fluorescent intensity of avidin, streptavidin, or CaptAvidin bound to either biotinylated or desthiobiotinylated scaffolds with increasing biotin densities (*n* = 10). Statistical significance determined using Ordinary two-way ANOVA followed by Tukey’s multiple comparison test. *p < 0.05, **p < 0.01, ***p < 0.001, ****p < 0.0001. For (*E*-O) release was measured over 14 days. Release profiles as depicted were ended when no mass of IL-4 was detected in at least 60 % of samples in that group. (E-G) Release of bIL-4 from biotinylated scaffolds bound streptavidin, avidin, or CaptAvidin over 7 days with scaffolds with adsorbed bIL-4 acting as a control. (H) Percent of bIL-4 released from biotinylated scaffolds bound with either avidin, streptavidin, or CaptAvidin at each timepoint, with 100 % representing all detectable bIL-4 released from the scaffold. (I–_K)_ Release of bIL-4 from desthiobiotinylated scaffolds bound streptavidin, avidin, or CaptAvidin over 7 days with scaffolds with adsorbed bIL-4 acting as a control. (L) Percent of bIL-4 released from desthiobiotinylated scaffolds bound with either avidin, streptavidin, or CaptAvidin at each timepoint, with 100 % representing all bIL-4 released from the scaffold. (M-O) Percent of bIL-4 released from either biotinylated or desthiobiotinylated scaffolds bound with either streptavidin, avidin, or CaptAvidin at each timepoint, with 100 % representing all detectable bIL-4 released from the scaffold. Data represented as mean +/− SD with *n* = 4–5. MFI, mean fluorescent intensity. FME, fold molar excess. Strep, streptavidin. Capt, CaptAvidin. biotin, biotinylated scaffolds. Desthio, desthiobiotinylated scaffolds.

Next, we prepared modified porous gelatin scaffolds and characterized how biotin or desthiobiotin affected the binding of avidin variants (avidin, streptavidin, or CaptAvidin) and subsequent release of biotinylated IL-4 (bIL-4) (Fig. 3A). Increasing either the biotinylation or desthiobiotinylation reagent concentration significantly increased binding of avidin and streptavidin in a dose dependent manner, as measured by increasing fluorescent intensities of the scaffolds, while binding of CaptAvidin did not differ based on the dose of the biotinylation or desthiobiotinylation reagent (Fig. 3B–D). However, there were some differences in binding of avidin variant on biotinylated scaffolds compared to desthiobiotinylated scaffolds. For example, streptavidin showed slightly but significantly more binding to biotinylated scaffolds compared to desthiobiotinylated scaffolds at mid biotinylation (1 FME), while CaptAvidin and avidin only showed differences at high biotinylation (10 FME) (Suppl. Fig. S6). Overall, these results show that binding of avidin variants could be controlled by changing the concentration of the biotinylation or desthiobiotinylation reagent.

Release of bIL-4 from modified scaffolds over 14 days in PBS was characterized using the 1-FME density of biotin or desthiobiotin with avidin variants and bIL-4, in comparison to corresponding biotinylated or desthiobiotinylated scaffolds with adsorbed bIL-4 (i.e., no avidin variants). Biotinylated IL-4 was released from all modified scaffolds. Streptavidin modified scaffolds had detectable release of bIL-4 for the longest time, over 7 days, with no detectable release beyond that time point from any scaffolds (Fig. 3E–O, Suppl. Fig. S7). While bIL-4 was released within 6 h from the adsorbed control, release was extended up to 7 days when streptavidin was present, up to 4 days when avidin was present, and up to 24 h when CaptAvidin was present (Fig. 3E–G). To compare release profiles between avidin variants, we analyzed fractional release profiles by dividing mass released at each time point by the total mass released after 7 days. bIL-4 released more slowly from biotinylated scaffolds bound with either streptavidin or avidin compared to scaffolds with adsorbed bIL-4 or bound with CaptAvidin (Fig. 3H).

For desthiobiotinylated scaffolds, only those bound with streptavidin extended release of bIL-4 compared to adsorbed controls while avidin or CaptAvidin did not (Fig. 3I–K). When comparing release profiles for desthiobiotinylated scaffolds, streptavidin clearly extended release of bIL-4 (Fig. 3L). When streptavidin and avidin were used with biotinylated scaffolds, release of bIL-4 was distinctly detected for longer than when scaffolds were conjugated with desthiobiotin, however when CaptAvidin was used, there were no differences in release duration (Fig. 3M–O). This extension in release of bIL-4 suggests that biotin-streptavidin and biotin-avidin affinity interactions slowed release of bIL-4 from the scaffolds.

### 3.3. Excess free biotin accelerates release of bIL-4 from modified scaffolds

**Fig. 4.**
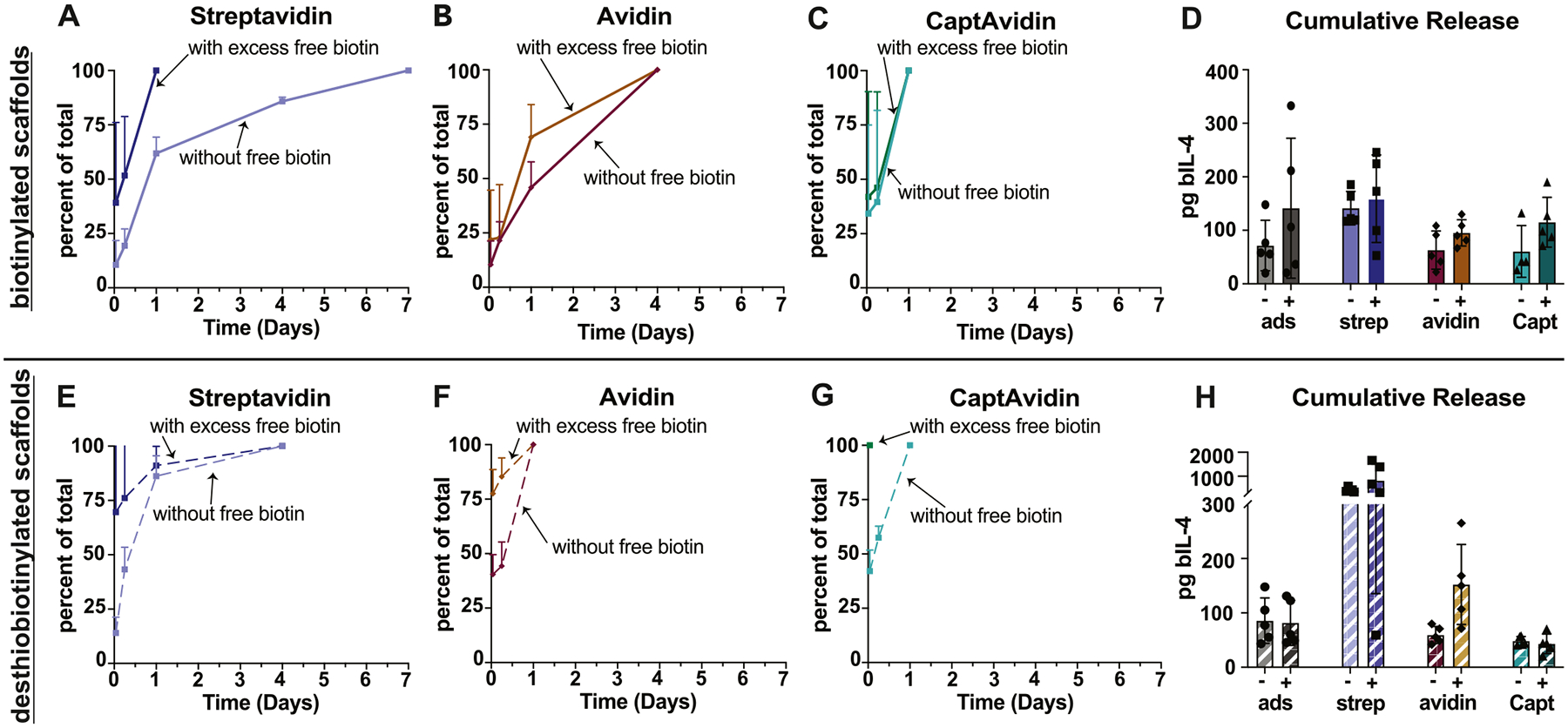
Release of biotinylated IL-4 (bIL-4) from modified gelatin scaffolds in presence or absence of free biotin. For (A-H) release was measured over 14 days and excess free biotin was maintained in the release media. Release profiles as depicted were ended when no mass of IL-4 was detected in at least 60 % of samples in that group. (A - C) Percent of bIL-4 released from biotinylated scaffolds bound with streptavidin, avidin, or CaptAvidin either in the absence (without free biotin) or presence (with free biotin) of 10 mM free biotin in solution at each timepoint, with 100 % representing all detectable bIL-4 released from the scaffold. (D) Total mass of bIL-4 released over 7 days for biotinylated scaffolds bound with streptavidin, CaptAvidin, or avidin either in the absence (−) or presence (+) of 10 mM free biotin in solution. (E - G) Percent of bIL-4 released from desthiobiotinylated scaffolds bound with streptavidin, avidin, or CaptAvidin either in the absence (without free biotin) or presence (with free biotin) of 10 mM free biotin in solution at each timepoint, with 100 % representing all detectable bIL-4 released from the scaffold. (H) Total mass of bIL-4 released over 14 days for desthiobiotinylated scaffolds bound with streptavidin, CaptAvidin, or avidin either in the absence (−) or presence (+) of 10 mM free biotin in solution. Data represented as mean +/− SD with *n* = 5. ads, adsorbed. Strep, streptavidin. Capt, CaptAvidin.

To confirm that the slower release of bIL-4 from biotinylated scaffolds compared to the adsorbed control resulted from biotin-avidin affinity interactions, we carried out release studies for 14 days, maintaining excess free biotin in the media. It has been previously demonstrated that biotinylated molecules have lower binding affinities to avidin and avidin variants compared to free biotin, such that excess free biotin outcompetes biotinylated molecules and displaces the larger molecule [12,43,44,58]. For biotinylated scaffolds bound with streptavidin or avidin, excess free biotin accelerated the release of bIL-4 but did not affect scaffolds bound with CaptAvidin (Fig. 4A–C, Suppl. Fig. S8). The total amount released was slightly but not significantly higher in the presence of free biotin compared to its absence (Fig. 4D). These trends were similar, but less pronounced, with the desthiobiotinylated scaffolds (Fig. 4E – H). In contrast, the presence of free biotin did not accelerate release of bIL-4 from adsorbed controls (Suppl. Fig. S9). Overall, the acceleration of release of bIL-4 in the presence of free biotin confirms that biotin-avidin interactions slow the release of bIL-4 from modified scaffolds in the absence of free biotin.

### 3.4. Biotin-streptavidin mediated presentation of bIL-4 promotes reparative phenotype in primary human macrophages

**Fig. 5.**
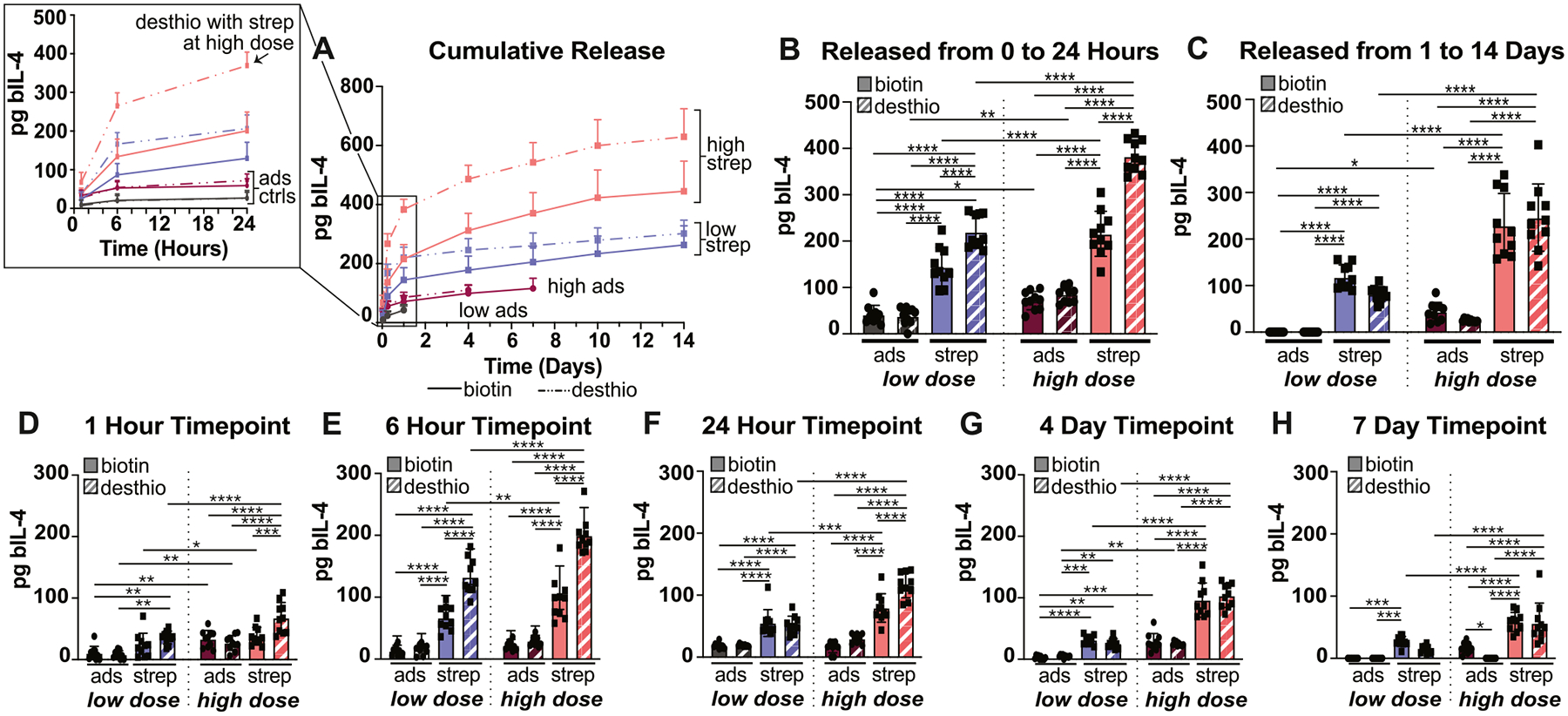
Release of biotinylated IL-4 (bIL-4) from modified gelatin scaffolds at a low and high dose of bIL-4. (A) Release of bIL-4 at a low (400 ng) or high (2000 ng) dose of bIL-4 from either biotinylated or desthiobiotinylated scaffolds bound with streptavidin over 14 days with scaffolds with adsorbed bIL-4 acting as a control. (B) Total mass of bIL-4 released from scaffolds within the first 24 h of the study. (C) Total mass of bIL-4 released from scaffolds between 24 h and 14 days. (D–H) Mass of bIL-4 released at select timepoint from modified scaffolds at a low or high dose of bIL-4. Data represented as mean +/− SD with *n* = 10. Statistical significance determined using Ordinary two-way ANOVA followed by Tukey’s multiple comparison test. *p < 0.05, **p < 0.01, ***p < 0.001, ****p < 0.0001. low, low dose bIL-4 (400 ng). high, high dose bIL-4 (2000 ng). ads, adsorbed. Ctrls, controls. Strep, streptavidin. Biotin, biotinylated scaffold. Desthio, desthiobiotinylated scaffold.

Before interrogating the effect of modified scaffolds on macrophage phenotype, we conducted release studies with a higher dose of bIL-4 loaded onto the modified scaffolds to increase likelihood of influencing macrophage behavior. Previous work has demonstrated that as little as 1 ng/mL of IL-4 can stimulate macrophages [59] but typically 40 ng/mL is used to promote a reparative macrophage phenotype in cell culture. We also focused on biotinylated and desthiobiotinylated scaffolds bound with streptavidin, since those formulations had the greatest mass of bIL-4 released. A five-fold increase in dose resulted in twice as much bIL-4 released from biotinylated scaffolds and a three-fold increase in bIL-4 released from desthiobiotinylated scaffolds (Fig. 5). Within the first 24 h of the study, both biotinylated and desthiobiotinylated scaffolds at both the low (400 ng bIL-4/scaffold) and high (2000 ng bIL-4/scaffold) doses released significantly more bIL-4 compared to adsorbed controls, with desthiobiotinylated scaffolds bound with streptavidin and the high dose of bIL-4 releasing significantly more than all other formulations (Fig. 5A and B). After the first 24 h, scaffolds with streptavidin continued to release significantly more bIL-4 than their respective controls with detectable release for up to 14 days (Fig. 5C). These trends held true over the entire release study, with scaffolds bound with streptavidin releasing significantly more bIL-4 over more days than respective controls (no streptavidin bound) (Fig. 5D–H).

**Fig. 6.**
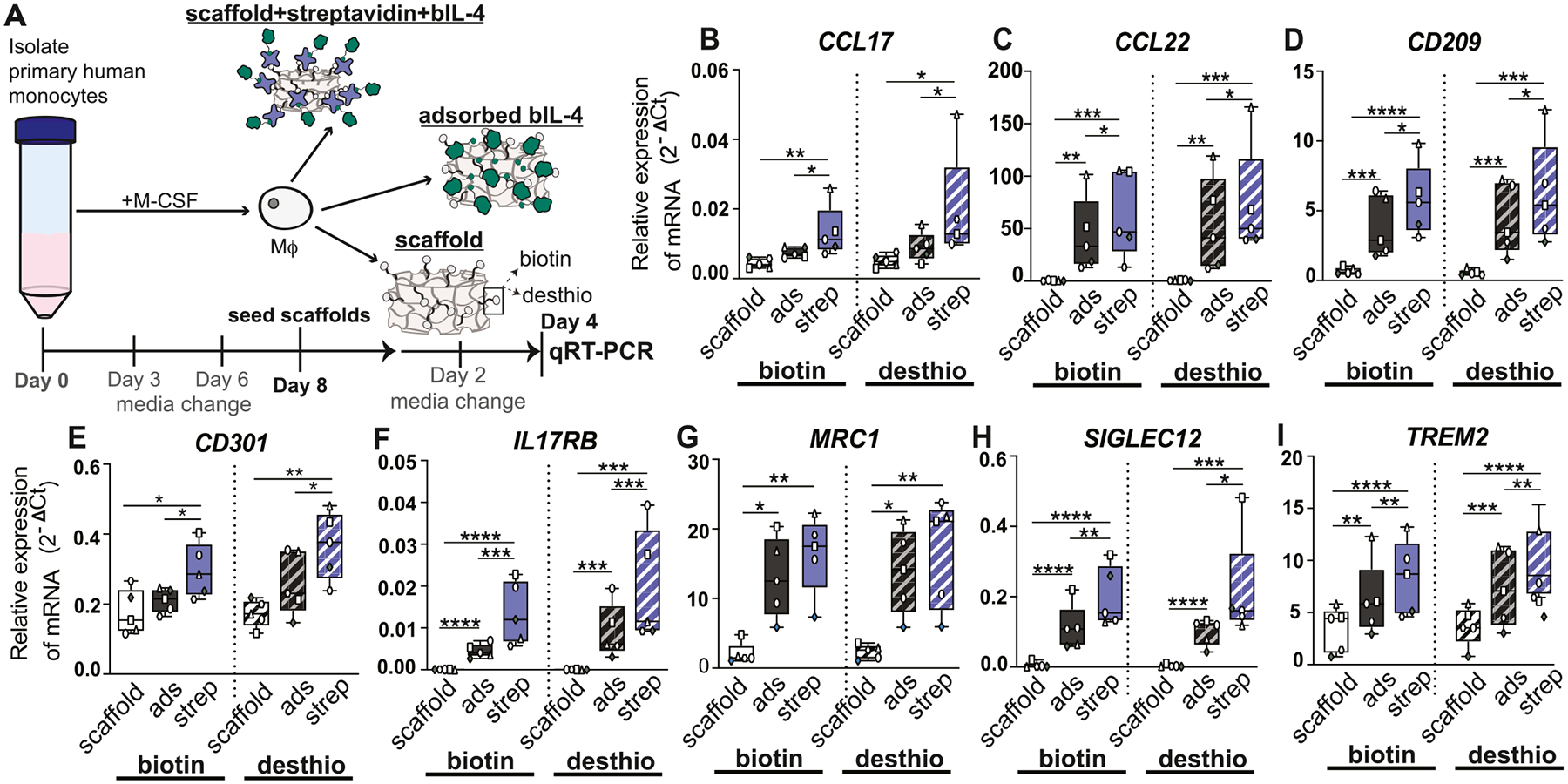
Expression of markers of IL-4 mediated polarization by qRT-PCR. (A) Experimental design. (B - I) Relative gene expression from macrophages seeded onto biotinylated scaffolds (negative control), biotinylated scaffolds with bIL-4 (adsorbed control), biotinylated scaffolds with streptavidin and bIL-4 (strep group), desthiobiotinylated scaffolds (negative control), desthiobiotinylated scaffolds with bIL-4 (adsorbed control), or desthiobiotinylated scaffold with streptavidin and bIL-4 (strep group). Data represented as mean +/− SD with n = 5. 4 male (white) and 1 female (gray) with each donor represented by a unique symbol. Statistical significance determined using RM two-way ANOVA with the Geisser-Greenhouse correction, followed by Tukey’s multiple comparison test. *p < 0.05, **p < 0.01, ***p < 0.001, ****p < 0.0001. qRT-PCR, quantitative real-time polymerase chain reaction. Mϕ, macrophage. Scaffold, indicates either a biotinylated or desthiobiotinylated scaffold.

Finally, we investigated how biotin-streptavidin-bIL-4 modified scaffolds influenced the phenotype of primary human macrophages from 5 donors using the scaffolds prepared with the high dose of bIL-4 (Fig. 6). We measured expression of genes associated with the IL-4-stimulated phenotype (sometimes called M2a) as well as other genes associated with different phenotypes of macrophages, including pro-inflammatory phenotypes, determined from a previous RNASeq analysis of primary human macrophage phenotypes [60]. Primary human macrophages were seeded onto biotinylated or desthiobiotinylated scaffolds with bound streptavidin and b-IL4 and cultured for 4 days with a media change at day 2 (Fig. 6A). All eight of the IL4-phenotype-associated genes (*CCL17, CCL22, CD209, CD301, IL17RB, SIGLEC12*, and *TREM2*) were upregulated by macrophages cultured on scaffolds modified with streptavidin and bIL-4 compared to when the bIL-4 was simply adsorbed to the scaffold, for both biotinylated and desthiobiotinylated scaffolds, although this effect was not significant for *MRC1* (Fig. 6B–F, H, and I). Finally, we evaluated markers associated with inflammatory phenotypes (*CCR7, CD80, CXCL9, IL1B, IL4R, IL13RA1*, and *VEGFA*,) and found that they were generally unaffected in any group compared to the unmodified scaffold controls (Suppl. Fig. S10A – S10C, S10E – S10G, and S10J). *MERTK*, which is involved in efferocytosis of apoptotic cells and is typically downregulated by IL-4, was significantly downregulated in all groups containing bIL-4 compared to controls (Fig. S10H). Interestingly, of all 19 genes evaluated, only one gene (*IL13RA1)* was expressed at different levels between macrophages cultured on biotinylated vs. desthiobiotinylated scaffolds bound with streptavidin and bIL-4 (Suppl. Fig. S11). Taken together, these results show that increased reparative macrophage polarization when bIL-4 is presented via biotin-streptavidin interactions compared to bIL-4 adsorbed to the scaffold, without influencing inflammatory phenotypes.

## 4. Discussion

This study shows that biotin-streptavidin interactions can be leveraged as a controlled affinity-based release platform to deliver an immunomodulatory cytokine and drive reparative polarization of macrophages in response to a model biomaterial. We demonstrated spontaneous release of biotinylated IL-4 (bIL-4), with detectable levels releasing up to 14 days from porous gelatin scaffolds depending on biotin and avidin variant and especially at higher doses of bIL-4. We also found that biotin-streptavidin-presented bIL-4 induced stronger gene expression of IL-4 driven genes in macrophages compared to scaffolds with adsorbed bIL-4. Taken together, these findings underscore the utility of the biotin-streptavidin system as a controlled delivery system. This system has the potential to modify complex biomaterials to impart immunomodulatory activity by influencing the local macrophage response. Example biomaterials expected to benefit from this strategy include complex implants such as prosthetic joints, stents, electrode arrays, glucose sensors, catheters, and engineered tissues, among others.

Previously, we demonstrated that bIL-4 can be attached to decellularized bone [12] and to porous gelatin scaffolds [45] via avidin variants, however it was not clear in those studies whether biotin-avidin affinity interactions influenced the rate of release of bIL-4. In the present study, we showed that bIL-4 was released from biotinylated scaffolds bound with avidin variants at a slower rate than simple desorption but faster than what would be expected based on the dissociation constant between free biotin and streptavidin or avidin. The addition of free biotin also accelerated release of bIL-4 from both biotinylated and desthiobiotinylated scaffolds, further demonstrating affinity-based release. These results agree with previous reports that directly investigated binding of free biotin or biotin conjugates to avidin variants [61] and other studies that used free biotin to outcompete and therefore induce release of biotin conjugates from biomaterials [42,44,62]. Deng et al. reported release of biotinylated exosomes from a biotin-avidin modified gelatin methacrylate hydrogel over a period of 7 days without introducing free biotin into the system [63]. We have built on these findings, and our own previous work, to show that spontaneous release of a biotinylated protein occurs at a slower rate than desorption-based release. Moreover, this study demonstrated even faster release by using desthiobiotin on the scaffolds. It has been well reported that desthiobiotin has a higher dissociation constant from both avidin and streptavidin compared to biotin [41,64,65], but to our knowledge no published reports have demonstrated its utility for controlling release of biotinylated proteins. In the present study, we showed accelerated release of bIL-4 from scaffolds conjugated with desthiobiotin compared to scaffolds conjugated with biotin, as expected. Together, these results demonstrate the utility of biotin or desthiobiotin complexed with avidin or streptavidin to achieve controlled release of proteins from biomaterials. These results are important since biotin (and presumably desthiobiotin) can be attached to a wide variety of molecules, including proteins [12] and cells [34,35] without compromising their functions, thus enabling controlled release of a wide variety of bioactive factors without complex modifications.

Biotin-avidin interactions have been used for targeted delivery in vivo in humans, as in delivery of radioimmunotherapy to tumors [66,67]. In these studies, the authors used a two-step method, in which they first administered biotinylated nanocarriers loaded with a chemotherapeutic that targeted tumor cells, then cleared unbound biotinylated chemotherapeutic nanocarriers with an injection of avidin [67]. These studies not only highlighted the specificity of the biotin-avidin interaction, but also the safety of this therapeutic drug delivery system in human patients. In a murine model of liver fibrosis, Jain et al. demonstrated the use of biotin-avidin interactions to create siRNA nano-complexes to protect, carry, and present siRNA to hepatic stellate cells to silence a key gene indicated in liver fibrosis [68]. Here, we expanded on that idea of presentation and examined how biotin-streptavidin presentation of bIL-4 affected primary human macrophages, a complex and highly plastic cell type. Biotin-streptavidin scaffolds upregulated IL-4 driven markers significantly more than simply adsorbing bIL-4 alone on the scaffold and did not significantly upregulate non-IL-4 driven markers. These effects were repeated across 5 human donors. While gene expression does not always translate to the protein level, the data presented here suggests the feasibility of biotin-avidin technology as an immunomodulatory tool.

Surprisingly, RNASeq did not reveal any differentially expressed genes when macrophages were treated with avidin, or streptavidin, compared to untreated macrophages. Previously, avidin was investigated as both an adjuvant and a protein carrier for vaccines [46,47]. While antibody titers in response to the antigen were modestly elevated in the presence of avidin, it was ultimately phased out as an adjuvant. Nonetheless, the fact that there was a slight adaptive immune response led us to wonder if avidin would also influence macrophage phenotype, especially since macrophages are responsive to a wide range of stimuli. While we observed a significant impact of CaptAvidin on macrophages, almost no change was observed in avidin-treated macrophages, and only a very modest response to streptavidin treatment was measured. CaptAvidin is avidin-derived but is modified so that a tyrosine in the biotin-binding cavity is nitrated [39]. The process to nitrate that tyrosine group or the resulting structure of the modified avidin may have stimulated macrophages to the gene expression levels we reported here. The GO pathways influenced by CaptAvidin were not obviously linked to immune cell function, and there were only 15 DEGs, but we cannot exclude the possibility that contamination of very low levels of endotoxin or other impurities could have influenced the results for any of the avidin variants tested, especially since macrophages are very sensitive and upregulate inflammatory pathways in response to doses of lipopolysaccharide (LPS) as low as 0.01 ng/mL [69]. Additionally, we only saw an influence of biotin on macrophages at the high dose used in some biological processes and cellular communication, which agrees with biotin’s role in metabolism [70] and as a coenzyme [71]. Overall, these results suggest that while avidin variants may be slightly immunogenic in vivo, streptavidin and avidin have minimal effects on macrophage phenotype in vitro, at least in terms of transcriptomic analysis. However, future studies should include more extensive characterization of the macrophage response in vitro, including longer time points and protein-level and functional analysis. Finally, the next step should be to determine if these in vitro results transfer to the in vivo environment for beneficial effects on biomaterial outcomes.

## 5. Conclusions

We defined modification parameters that not only achieved spontaneous release of bIL-4 from a model biomaterial but also promoted a reparative macrophage phenotype using biotin-streptavidin interactions. RNASeq analysis of avidin, its variants, and biotin demonstrated how this affinity pair affects macrophage phenotype. We also demonstrated slower release of bIL-4 from a porous gelatin scaffold as a function of biotin variants and avidin variants compared to desorption-based release. Finally, we highlighted how either a biotinylated or desthiobiotinylated scaffold bound with streptavidin and bIL-4 promoted greater reparative macrophage polarization compared to either an unmodified scaffold control or a scaffold with adsorbed bIL-4. Future work should utilize this platform technology to explore if these effects on macrophages are observed in vivo, as well as apply this platform to more complex biomaterials and different cytokines of interest for various clinical applications.

## Supplementary Material

Supplementary Material

## Data Availability

RNAseq data will be deposited into GEO

## Appendix A. Supplementary data

Supplementary data to this article can be found online at https://doi.org/10.1016/j.jconrel.2025.113943.
