# Supplementary material for: Affinity-based controlled release of interleukin-4 from scaffolds via biotin-streptavidin interactions for immunomodulation
Source: J Control Release. Author manuscript; Available in PMC 2026 Apr 27. (PMC13112467; doi:10.1016/j.jconrel.2025.113943)
Supplement: Supplementary Material [file NIHMS2163143-supplement-Supplementary_Material.pdf]

Supporting Information for:
Affinity-based controlled release of interleukin-4 from biotin-streptavidin modified scaffolds for immunomodulation

*Victoria A. Nash[a], Juan F. Cortes[a], Phoebe E. Chua[a], Kara L. Spiller[a]**

[a]School of Biomedical Engineering, Sciences and Health Systems, Drexel University, Philadelphia PA 19104

*Corresponding author:
Kara L. Spiller, Ph.D. School of Biomedical Engineering, Science, and Health Systems, Drexel University. 3141 Chestnut Street. Bossone 718. Philadelphia, PA 19104. spiller@drexel.edu. Phone: +1 215 571 3822.

Table S1: Donor information for frozen peripheral blood mononuclear cells purchased from the Human Immunology Core at the University of Pennsylvania, Philadelphia, PA.

Donor ID	Blood Type	Race	Gender	Age
ND578	unknown	unknown	male	31
ND561	unknown	unknown	male	27
ND601	unknown	unknown	male	26
ND582	unknown	unknown	female	52
ND587	unknown	unknown	male	25
ND518	unknown	unknown	female	39
ND502	unknown	unknown	female	55

Table S2: Donor information for monocytes isolated from leukopaks purchased from the New York Blood Center, New York, NY.

Donor ID	Blood Type	Race	Gender	Age
9000 W04702329105300C	AB+	White	male	65
9000 W04702329106000E	A+	White	male	36
9000 W04702327775500V	B+	Asian	male	67
9000 W04702326821600R	B+	Black	female	64
9000 W047023289270009	B+	Asian	male	26

Table S3: List of primers used for qRT-PCR.

Oligo sequence (5' to 3')	Oligo name
TCCACAACACAGTAATTGGTCAC	VPS29_FW
AGATTCTTTCTCCACATGCACT	VPS29_RW
TCTCCCTACCCCTTCAACCT	UBE2D2_FW
TGGAAGAGAGAAAGAAGGGCTG	UBE2D2_RW
TTCCCTTTGGGCGGATTGTT	HPRT1_FW
CACTCAATAGTGCTGTGGTTTAAGA	HPRT1_RW
CCTTTGAGGAGCACTGGAGG	SDHA_FW
AGTGCCAACGTCCACATAGG	SDHA_RW
GTGTGGTCAGCCTCTCTCAC	TIMP3_FW
CACTCAGCCTTCACCCAAGT	TIMP3_RW
ACGTGTGCACCTACCTCAA	MRC1_FW
CCTGGGCTTGACTGACTGTT	MRC1_RW
GCGGGAAGAAGAGACACACA	PDGFB_FW
CACAAGTCCACGTGTCAGGA	PDGFB_RW
AGCAGTGTCTGTAAAAGAGCCT	IL1B_FW
AGAGCACACCAGTCCAAATTGA	IL1B_RW
GCGTGATCAAAGCCACACTC	CCR7_FW
AGCTGAGTGCATGTCATCCC	CCR7_RW
CCCCATGAGGTACAGCAGAA	CD80_FW
TCCTTGACTACTGCTTTGACGT	CD80_RW
TTGGTGCTACTGTTTATCCGT	VEGFA_FW
TGCACTAGAGACAAAGACGTGA	VEGFA_RW
CTCTGGCGTAGAGCTATCACT	MERTK_FW
AGGCTGGGTTGGTGAAAACA	MERTK_RW
GAGTGCTGCCTGGAGTACTT	CCL17_FW
GCAGTCCTCAGATGTCTGGT	CCL17_RW
AATGGCTGGAACGACGACAAA	CD209_FW
CAGGAGGCTGCGGACTTTTT	CD209_RW
ATGTCGCTCGTGCTGCTAAG	IL17RB_FW
AGCCACATTGAACGGTCGG	IL17RB_RW
CCAGTAGTGAGAAAGGGTCGC	CXCL9_FW
AGGGCTTGGGGCAAATTGTT	CXCL9_RW
CACCTATGCAGTCAACATTTGGA	IL4R_FW
GATGCGGAGGGAGGGTTCTA	IL4R_RW
ACAACCTGAGCTACATGAAGTG	IL13RA1_FW
GGCTTCTGTGCCAATAGTAGAG	IL13RA1_RW
GCTCCGCATACACCTGGATG	CLEC10A_FW
GCCGGTCGCATAGTCTGTTC	CLEC10A_RW
GGAAGGGCAAGCTATGAAAGG	FGF14_FW
TGGTTCTCGGTACATGGCAAC	FGF14_RW
GGGCAATGGATTGGTCATCCT	CXCR4_FW
TGCAGCCTGTACTTGTCCG	CXCR4_RW
GGTCAGCACGCACAACTTG	TREM2_FW
CGCAGCGTAATGGTGAGAGT	TREM2_RW
GGCATCCAGCTCAGTGTCAT	CCL22_FW
CGTCCTTCTAGTGCCTGTGG	CCL22_RW
GGATACAGGCATGGAGGACG	SIGLEC12_FW
GTCATCTGCCGGGGATTCAA	SIGLEC12_RW

Table S4: Differentially expressed genes for macrophages treated with CaptAvidin compared to untreated macrophages.

Gene Name	Gene ID	adjusted pvalue	log2FoldChange	Gene Biotype
SLC5A3	ENSG00000198743	4.38E-11	1.384911827	protein_coding
CXCL8	ENSG00000169429	4.38E-11	2.248143329	protein_coding
ZC3H12C	ENSG00000149289	1.77E-05	0.97497763	protein_coding
TNFAIP6	ENSG00000123610	0.000132892	1.747006287	protein_coding
SOD2	ENSG00000112096	0.000335208	1.15368776	protein_coding
CRIM1	ENSG00000150938	0.000830701	1.166136273	protein_coding
HMOX1	ENSG00000100292	0.003508172	-0.56347386	protein_coding
MGST3	ENSG00000143198	0.004217272	-0.564761655	protein_coding
PTPN13	ENSG00000163629	0.004217272	1.168072647	protein_coding
ARNTL2	ENSG00000029153	0.004217272	0.956398879	protein_coding
FABP5	ENSG00000164687	0.004217272	-0.846112191	protein_coding
IER3	ENSG00000137331	0.004217272	0.748549221	protein_coding
SELENOW	ENSG00000178980	0.004217272	-0.742605023	protein_coding
BLVRB	ENSG00000090013	0.004217272	-0.792148116	protein_coding
DYRK2	ENSG00000127334	0.004608702	0.529811045	protein_coding
FER	ENSG00000151422	0.004608702	0.816285721	protein_coding
NFE2L2	ENSG00000116044	0.004608702	0.530929504	protein_coding
PLIN2	ENSG00000147872	0.004608702	-0.530639034	protein_coding
S100A11	ENSG00000163191	0.006719119	-0.541946683	protein_coding
TRAF1	ENSG00000056558	0.006719119	0.739037014	protein_coding
NBN	ENSG00000104320	0.006953651	0.798530925	protein_coding
NFKBIZ	ENSG00000144802	0.006971411	1.424863177	protein_coding
FABP5P7	ENSG00000234964	0.006971411	-0.859010296	processed_pseudogene
FAM162A	ENSG00000114023	0.006971411	-0.60938582	protein_coding
RPSA	ENSG00000168028	0.007952229	-0.594494835	protein_coding
C1orf162	ENSG00000143110	0.00812053	-0.499214223	protein_coding
SDS	ENSG00000135094	0.008354191	-1.064392323	protein_coding
ERGIC1	ENSG00000113719	0.009336362	-0.437091918	protein_coding
TREM2	ENSG00000095970	0.010354088	-0.733866977	protein_coding
STMN1	ENSG00000117632	0.010543693	-1.016103903	protein_coding
GPD1	ENSG00000167588	0.010543693	-1.158936076	protein_coding
NCOA2	ENSG00000140396	0.010543693	0.569190731	protein_coding
NCAPH	ENSG00000121152	0.010543693	-0.662778495	protein_coding
CTSL	ENSG00000135047	0.011907686	-0.745022234	protein_coding
BX679664.3	ENSG00000244716	0.01268854	-0.917813244	processed_pseudogene
AKR1B1	ENSG00000085662	0.01268854	0.794215888	protein_coding
ATP6V0D1	ENSG00000159720	0.01291995	-0.475785251	protein_coding
BHLHE41	ENSG00000123095	0.013493398	0.822809891	protein_coding
CHPF	ENSG00000123989	0.013493398	-0.685521145	protein_coding
BABAM1	ENSG00000105393	0.013493398	-0.568069779	protein_coding
FTL	ENSG00000087086	0.013493398	-0.539285374	protein_coding
HELZ2	ENSG00000130589	0.013493398	0.593327978	protein_coding
NUP188	ENSG00000095319	0.013493398	0.453346544	protein_coding
APOL3	ENSG00000128284	0.013493398	0.674559854	protein_coding
FTX	ENSG00000230590	0.01446222	0.921192714	lincRNA
SDF2L1	ENSG00000128228	0.015961089	-0.751335005	protein_coding
RMDN3	ENSG00000137824	0.016023683	-0.553025034	protein_coding
CLEC4E	ENSG00000166523	0.017136627	1.082581959	protein_coding
C12orf10	ENSG00000139637	0.017136627	-0.678078273	protein_coding

Table S4(continued).

Gene Name	Gene ID	adjusted pvalue	log2FoldChange	Gene Biotype
RPLP0	ENSG00000089157	0.017136627	-0.605823926	protein_coding
HMGN2	ENSG00000198830	0.017449756	-0.588197848	protein_coding
SLC1A3	ENSG00000079215	0.017766513	0.525379393	protein_coding
CTC1	ENSG00000178971	0.018047499	0.5858551	protein_coding
S100A6	ENSG00000197956	0.018047499	-0.701009385	protein_coding
RPL21P16	ENSG00000220842	0.018047499	-0.773814937	processed_pseudogene
HMGA1	ENSG00000137309	0.019490875	-0.409554637	protein_coding
MRPL37	ENSG00000116221	0.019490875	-0.386028899	protein_coding
TRAPPC2L	ENSG00000167515	0.020289726	-0.781805774	protein_coding
HIVEP1	ENSG00000095951	0.020359321	0.655617473	protein_coding
DNAH1	ENSG00000114841	0.020359321	1.037989236	protein_coding
ETHE1	ENSG00000105755	0.020359321	-0.685497699	protein_coding
CDK5	ENSG00000164885	0.020359321	-0.544281399	protein_coding
PCNT	ENSG00000160299	0.020359321	0.55031749	protein_coding
SNRPD2	ENSG00000125743	0.020359321	-0.671602227	protein_coding
CRABP2	ENSG00000143320	0.020794399	-0.726708274	protein_coding
ANAPC11	ENSG00000141552	0.021325057	-0.628590434	protein_coding
TNFAIP3	ENSG00000118503	0.021325057	0.836515593	protein_coding
C7orf50	ENSG00000146540	0.021325057	-0.7343967	protein_coding
SLC2A4RG	ENSG00000125520	0.021682686	-0.732014722	protein_coding
RHOC	ENSG00000155366	0.021941037	-0.594242327	protein_coding
PPDPF	ENSG00000125534	0.021941037	-0.748839496	protein_coding
MRPS15	ENSG00000116898	0.021978563	-0.390498817	protein_coding
NFKBIA	ENSG00000100906	0.021978563	0.737361798	protein_coding
ABHD14B	ENSG00000114779	0.023566038	-0.500922575	protein_coding
BLOC1S1	ENSG00000135441	0.023566038	-0.835556423	protein_coding
EPS8L2	ENSG00000177106	0.023566038	-0.943807613	protein_coding
SLC2A13	ENSG00000151229	0.024049838	0.495122643	protein_coding
CTSD	ENSG00000117984	0.024121431	-0.616739981	protein_coding
PIK3R5	ENSG00000141506	0.024121431	0.378654296	protein_coding
CORO1B	ENSG00000172725	0.024701953	-0.445609388	protein_coding
PSD3	ENSG00000156011	0.024701953	0.561883735	protein_coding
PPIB	ENSG00000166794	0.027445613	-0.45066593	protein_coding
CSTA	ENSG00000121552	0.028012913	-0.693706229	protein_coding
DDOST	ENSG00000244038	0.030156315	-0.327421175	protein_coding
ARF1	ENSG00000143761	0.030156315	-0.326922887	protein_coding
SDC3	ENSG00000162512	0.030472707	-0.652401789	protein_coding
GAS2L3	ENSG00000139354	0.030617174	0.609592137	protein_coding
MYL6	ENSG00000092841	0.030886204	-0.770564183	protein_coding
PARP14	ENSG00000173193	0.030886204	0.501662618	protein_coding
GLIPR2	ENSG00000122694	0.030886204	-0.448679922	protein_coding
MFAP3	ENSG00000037749	0.031540227	0.715103565	protein_coding
ID2	ENSG00000115738	0.031540227	-0.683101039	protein_coding
IDH2	ENSG00000182054	0.031540227	-0.358289936	protein_coding
UBA52	ENSG00000221983	0.032242206	-0.628027584	protein_coding
PLA2G15	ENSG00000103066	0.032749163	-0.556263743	protein_coding
FXYD5	ENSG00000089327	0.032749163	-0.53391541	protein_coding
RPS12	ENSG00000112306	0.032749163	-0.623367132	protein_coding
QDPR	ENSG00000151552	0.032749163	-0.407131095	protein_coding

Table S4 (continued).

Gene Name	Gene ID	adjusted pvalue	log2FoldChange	Gene Biotype
ICK	ENSG00000112144	0.032749163	0.603875664	protein_coding
PTPRJ	ENSG00000149177	0.033070134	0.452354451	protein_coding
CCDC82	ENSG00000149231	0.033070134	0.452900959	protein_coding
TNIK	ENSG00000154310	0.033178759	0.57291703	protein_coding
PSTPIP2	ENSG00000152229	0.034187834	0.699459949	protein_coding
MZT2B	ENSG00000152082	0.034187834	-0.752073675	protein_coding
H2AFZ	ENSG00000164032	0.0344732	-0.493056236	protein_coding
ANXA6	ENSG00000197043	0.0344732	-0.650026601	protein_coding
SDSL	ENSG00000139410	0.0344732	-0.632001456	protein_coding
TMEM37	ENSG00000171227	0.034906169	-1.296211249	protein_coding
UBL5	ENSG00000198258	0.035114872	-0.706755138	protein_coding
NPC2	ENSG00000119655	0.035869941	-0.44453072	protein_coding
CSTB	ENSG00000160213	0.037755844	-0.567311421	protein_coding
PRKD3	ENSG00000115825	0.037755844	0.486719532	protein_coding
JAG1	ENSG00000101384	0.037755844	0.870773269	protein_coding
DNAJB1	ENSG00000132002	0.038027456	-0.34117777	protein_coding
CLTB	ENSG00000175416	0.038363228	-0.57079787	protein_coding
RASGRP3	ENSG00000152689	0.038363228	0.378189818	protein_coding
ZMAT2	ENSG00000146007	0.038363228	-0.447714759	protein_coding
PLXNA1	ENSG00000114554	0.038368141	0.531192163	protein_coding
RPLP0P6	ENSG00000213553	0.039413746	-0.771744868	processed_pseudogene
CCDC59	ENSG00000133773	0.040079255	-0.703398889	protein_coding
RPS3	ENSG00000149273	0.041980163	-0.578400373	protein_coding
CLIC1	ENSG00000213719	0.04201822	-0.506386657	protein_coding
RPLP1	ENSG00000137818	0.042470135	-0.553775566	protein_coding
TBC1D9	ENSG00000109436	0.042470135	0.416698855	protein_coding
MRTFB	ENSG00000186260	0.042470135	0.671564086	protein_coding
OSTC	ENSG00000198856	0.042470135	-0.432088673	protein_coding
ABHD17A	ENSG00000129968	0.042470135	-0.453984408	protein_coding
PCNX1	ENSG00000100731	0.042470135	0.601178434	protein_coding
CHST2	ENSG00000175040	0.042539941	0.76212582	protein_coding
SLC25A39	ENSG00000013306	0.042539941	-0.388957038	protein_coding
LMAN2	ENSG00000169223	0.042539941	-0.508519919	protein_coding
TRAK2	ENSG00000115993	0.042659149	0.380610647	protein_coding
VPS13C	ENSG00000129003	0.043021447	0.601090555	protein_coding
CHCHD2	ENSG00000106153	0.044186176	-0.546326353	protein_coding
CYP1B1	ENSG00000138061	0.045198673	0.689998429	protein_coding
DAD1	ENSG00000129562	0.04535415	-0.43252541	protein_coding
ARHGAP21	ENSG00000107863	0.04535415	0.560052786	protein_coding
SRM	ENSG00000116649	0.04535415	-0.466733926	protein_coding
RNF187	ENSG00000168159	0.04587881	-0.497047055	protein_coding
CCDC85B	ENSG00000175602	0.04587881	-0.793469899	protein_coding
ECI1	ENSG00000167969	0.04587881	-0.53183479	protein_coding
PSMA7	ENSG00000101182	0.04587881	-0.516452836	protein_coding
COX6B1	ENSG00000126267	0.04587881	-0.623530852	protein_coding
SOD1	ENSG00000142168	0.04587881	-0.471041616	protein_coding
HIST2H2BE	ENSG00000184678	0.04587881	0.809517567	protein_coding
ZNF318	ENSG00000171467	0.04587881	0.487220222	protein_coding
PPP4C	ENSG00000149923	0.045889623	-0.512047891	protein_coding

Table S4 (continued).

Gene Name	Gene ID	adjusted pvalue	log2FoldChange	Gene Biotype
LUCAT1	ENSG00000248323	0.046167334	0.706363971	antisense
FBXL15	ENSG00000107872	0.046455835	-0.610768937	protein_coding
HMGN1	ENSG00000205581	0.046455835	-0.317720541	protein_coding
CDK2AP2	ENSG00000167797	0.046455835	-0.505231017	protein_coding
HAMP	ENSG00000105697	0.046455835	-0.911349825	protein_coding
SLC2A6	ENSG00000160326	0.046612529	1.297294811	protein_coding
SLFN5	ENSG00000166750	0.047734065	0.50911466	protein_coding
TMC6	ENSG00000141524	0.047734065	-0.388691361	protein_coding
JTB	ENSG00000143543	0.048424219	-0.590433615	protein_coding
AVPI1	ENSG00000119986	0.048467926	-0.675669635	protein_coding
SLC9A9	ENSG00000181804	0.049213712	0.845549157	protein_coding
RPS5	ENSG00000083845	0.049386071	-0.66382557	protein_coding
ZNF518A	ENSG00000177853	0.049386071	0.589447064	protein_coding
IL1B	ENSG00000125538	0.049386071	1.966173272	protein_coding
MT-TC	ENSG00000210140	0.049386071	-0.78530702	Mt_tRNA
CHID1	ENSG00000177830	0.049386071	-0.468083381	protein_coding
PDXK	ENSG00000160209	0.049386071	-0.298714057	protein_coding
AP1S1	ENSG00000106367	0.049386071	-0.591662268	protein_coding
MLLT6	ENSG00000275023	0.049386071	0.563662609	protein_coding
LSM2	ENSG00000204392	0.049386071	-0.606746522	protein_coding
NDUFB2	ENSG00000090266	0.049386071	-0.595563323	protein_coding
SIL1	ENSG00000120725	0.049386071	-0.441958197	protein_coding
NCOA3	ENSG00000124151	0.049386071	0.41988566	protein_coding
ANP32B	ENSG00000136938	0.049386071	-0.463973648	protein_coding
ZNF142	ENSG00000115568	0.049386071	0.597827104	protein_coding
RPS18	ENSG00000231500	0.049697734	-0.630771915	protein_coding
NFAT5	ENSG00000102908	0.049697734	0.770602792	protein_coding
PMP22	ENSG00000109099	0.049926321	-0.8005253	protein_coding

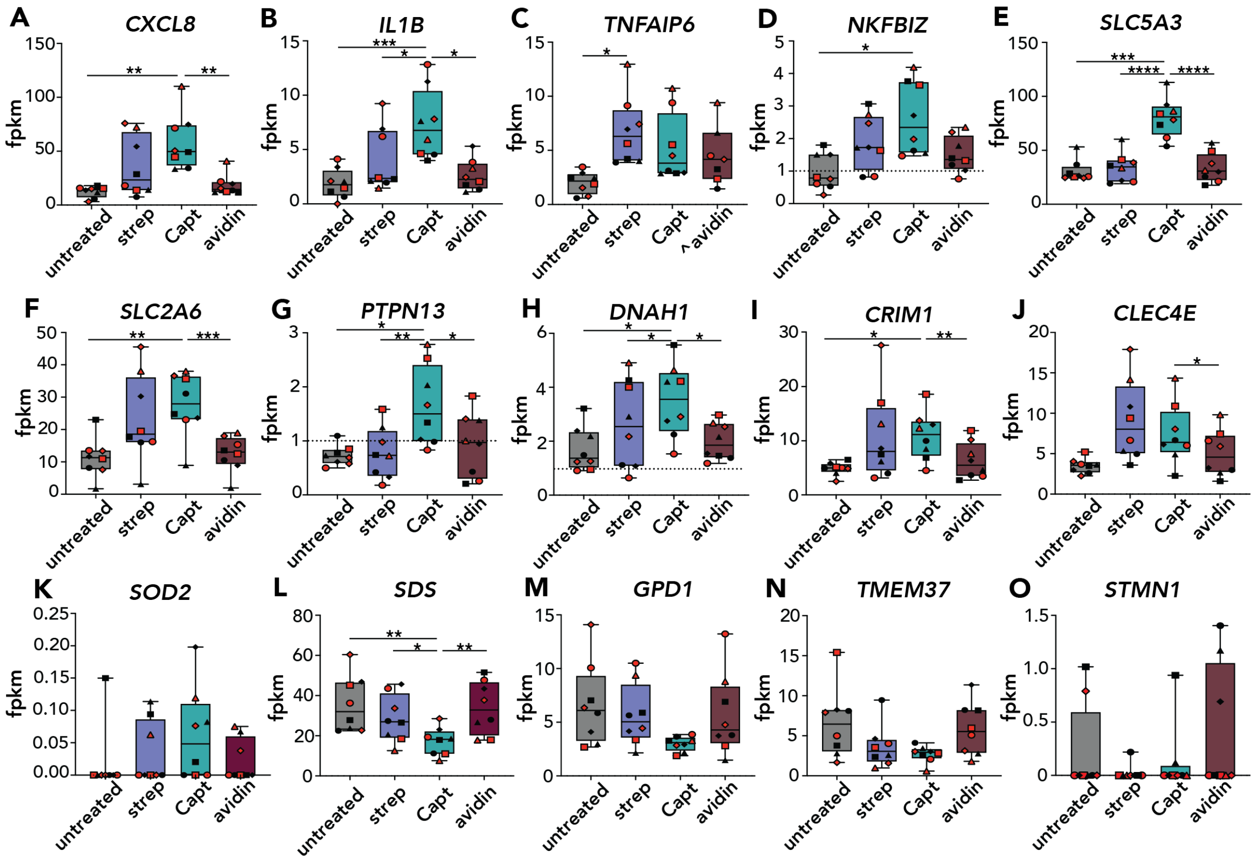

Figure S1: Differentially expressed genes (DEGs) with a p-adjusted<0.05 after the Benjamini-Hochberg (BH) procedure and an absolute log2(fold change)>1 that are protein coding for CaptAvidin.(**A – O**) Comparison of the effect of treatment with streptavidin, CaptAvidin, and avidin on M0 macrophages using DEGs identified using DESeq2 with a pvalue calculation model using a negative binomial distribution and the BH procedure for the false discovery rate calculation. Dotted line at 1 represents the threshold above which indicates expression above background. Data represented as mean +/- SD with n = 8 donors, 4 male and 4 female. Outliers were identified using the robust regression and outlier removal (ROUT) method with q=1%. ^ indicates that one outlier was removed from analysis for the avidin treatment for *TNFAIP6*. Statistical significance determined using RM One-way ANOVA or a mixed-effects analysis with Geisser-Greenhouse correction followed by Tukey's post-hoc multiple comparison test. *p<0.05, **p<0.01, ***p<0.001, ****p<0.0001. fpkm, fragments per kilobase of transcript sequence per million base pairs sequenced. strep, streptavidin. Capt, CaptAvidin.

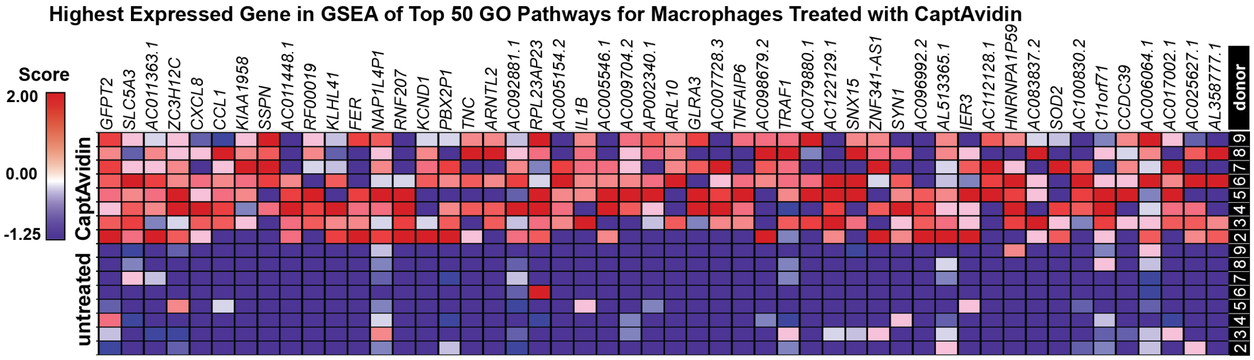

Figure S2: Heatmap of the most significantly enriched genes, determined by gene set enrichment analysis (GSEA) for the top 50 most significantly enriched GO pathways with CaptAvidin treatment for each donor. n = 8 donors, 4 male and 4 female with each donor represented by a unique symbol. strep, streptavidin. GSEA, gene set enrichment analysis.

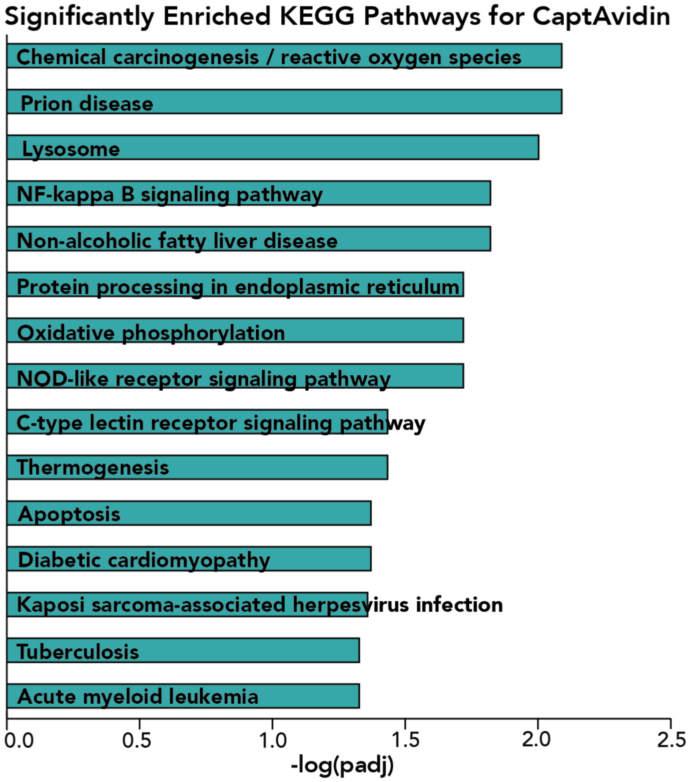

Figure S3: Significantly enriched Kyoto Encyclopedia of Genes and Genomes (KEGG) pathways for treatment with CaptAvidin. n = 8 donors, 4 male and 4 female with each donor represented by a unique symbol. Capt, CaptAvidin. KEGG, Kyoto Encyclopedia of Genes and Genomes

Table S5: Significantly enriched pathways from Kyoto Encyclopedia of Genes and Genomes (KEGG) analysis for the treatment of macrophages with CaptAvidin. KEGGID, KEGG ID.

KEGGID	Description	Gene Ratio	pvalue	padj	Count	Up	Down
hsa05208	Chemical carcinogenesis - reactive oxygen species	76/1214	2.56E-05	0.007780668	76	19	57
hsa05020	Prion disease	89/1214	4.82E-05	0.007780668	89	13	76
hsa04142	Lysosome	38/1214	8.83E-05	0.009510351	38	12	26
hsa04064	NF-kappa B signaling pathway	28/1214	0.00021	0.014433463	28	24	4
hsa04932	Non-alcoholic fatty liver disease	54/1214	0.00022	0.014433463	54	11	43
hsa04141	Protein processing in endoplasmic reticulum	44/1214	0.00039	0.018275573	44	9	35
hsa00190	Oxidative phosphorylation	56/1214	0.00045	0.018275573	56	4	52
hsa04621	NOD-like receptor signaling pathway	43/1214	0.00045	0.018275573	43	31	12
hsa04625	C-type lectin receptor signaling pathway	27/1214	0.001	0.035154141	27	20	7
hsa04714	Thermogenesis	71/1214	0.00109	0.035154141	71	16	55
hsa04210	Apoptosis	38/1214	0.0014	0.040754317	38	23	15
hsa05415	Diabetic cardiomyopathy	67/1214	0.00151	0.040754317	67	13	54
hsa05167	Kaposi sarcoma-associated herpesvirus infection	45/1214	0.00168	0.041804854	45	31	14
hsa05152	Tuberculosis	40/1214	0.00203	0.045048851	40	20	20
hsa05221	Acute myeloid leukemia	18/1214	0.00209	0.045048851	18	9	9

Table S6: Significantly enriched pathways from Disease Ontology (DO) analysis for the treatment of macrophages with CaptAvidin.

DO ID	Description	GeneRatio	pvalue	padj	Count	Up	Down
DOID:1342	congenital hypoplastic anemia	10/955	6.91E-05	0.02326773	10	4	6
DOID:9538	multiple myeloma	56/955	7.39E-05	0.02326773	56	30	26
DOID:8567	Hodgkin's lymphoma	17/955	9.19E-05	0.02326773	17	12	5
DOID:2349	arteriosclerosis	68/955	0.00019253	0.02326773	68	30	38
DOID:1936	atherosclerosis	66/955	0.00021341	0.02326773	66	30	36
DOID:007000[illegible]	myeloma	64/955	0.00023619	0.02326773	64	35	29
DOID:2348	arteriosclerotic cardiovascular disease	66/955	0.0002367	0.02326773	66	30	36
DOID:3070	malignant glioma	47/955	0.00024237	0.02326773	47	25	22
DOID:4960	bone marrow cancer	64/955	0.0003947	0.03368133	64	35	29

Table S7: Significantly enriched pathways from DisGeNET analysis for the treatment of macrophages with CaptAvidin. DisGeNETID, DisGeNET ID.

DisGeNETID	Description	GeneRatio	pvalue	padj	Count	Up	Down
C1260899	Anemia, Diamond-Blackfan	24/1755	2.23E-07	0.00056271	24	6	18
C0311375	Arsenic Poisoning	25/1755	2.43E-07	0.00056271	25	21	4
C0002886	Anemia, Macrocytic	15/1755	1.31E-06	0.00203172	15	5	10
C0220756	Niemann-Pick Disease, Type C	22/1755	1.53E-05	0.01768416	22	10	12
C0019829	Hodgkin Disease	100/1755	3.03E-05	0.02349417	100	55	45
C0006287	Bronchopulmonary Dysplasia	41/1755	3.48E-05	0.02349417	41	27	14
C0007138	Carcinoma, Transitional Cell	72/1755	3.55E-05	0.02349417	72	40	32
C1318544	M5b Acute differentiated monocytic leukemia	27/1755	7.03E-05	0.04077237	27	17	10
C1168327	High-Grade Prostatic Intraepithelial Neoplasia	24/1755	0.00010567	0.04427074	24	13	11
C0281966	reproductive system abnormality	9/1755	0.00011457	0.04427074	9	0	9
C0282606	Myomatous neoplasm	9/1755	0.00011457	0.04427074	9	6	3
C0744356	genital abnormal	9/1755	0.00011457	0.04427074	9	0	9

Table S8: Significantly enriched pathways from Reactome analysis for the treatment of macrophages with CaptAvidin. ReactomeID, Reactome pathway ID.

ReactomeID	Description	GeneRatio	pvalue	padj	Count	Up	Down
R-HSA-1799339	SRP-dependent cotranslational protein targeting to membrane	80/1406	3.93E-40	2.42E-37	80	0	79
R-HSA-156902	Peptide chain elongation	70/1406	5.89E-40	2.42E-37	70	0	69
R-HSA-192823	Viral mRNA Translation	70/1406	5.89E-40	2.42E-37	70	1	68
R-HSA-156842	Eukaryotic Translation Elongation	71/1406	1.62E-39	5.01E-37	71	0	70
R-HSA-72764	Eukaryotic Translation Termination	71/1406	5.85E-39	1.44E-36	71	0	70
R-HSA-72689	Formation of a pool of free 40S subunits	74/1406	5.97E-38	1.23E-35	74	0	73
R-HSA-2408557	Selenocysteine synthesis	70/1406	1.02E-37	1.80E-35	70	0	69
R-HSA-975956	Nonsense Mediated Decay (NMD) independent of the Exon Junction C	70/1406	1.10E-36	1.70E-34	70	0	69
R-HSA-156827	L13a-mediated translational silencing of Ceruloplasmin expression	76/1406	1.10E-35	1.51E-33	76	0	75
R-HSA-72613	Eukaryotic Translation Initiation	78/1406	1.42E-34	1.59E-32	78	0	77
R-HSA-72737	Cap-dependent Translation Initiation	78/1406	1.42E-34	1.59E-32	78	0	77
R-HSA-72706	GTP hydrolysis and joining of the 60S ribosomal subunit	75/1406	3.25E-34	3.35E-32	75	0	74
R-HSA-927802	Nonsense-Mediated Decay (NMD)	73/1406	2.05E-31	1.81E-29	73	1	71
R-HSA-975957	Nonsense Mediated Decay (NMD) enhanced by the Exon Junction Com	73/1406	2.05E-31	1.81E-29	73	1	71
R-HSA-168273	Influenza Viral RNA Transcription and Replication	79/1406	2.54E-31	2.09E-29	79	7	71
R-HSA-168255	Influenza Life Cycle	82/1406	6.49E-31	5.01E-29	82	7	74
R-HSA-168254	Influenza Infection	85/1406	1.33E-30	9.67E-29	85	7	77
R-HSA-9010553	Regulation of expression of SLITs and ROBOs	89/1406	2.15E-30	1.48E-28	89	1	87
R-HSA-2408522	Selenoamino acid metabolism	72/1406	9.81E-30	6.37E-28	72	0	71
R-HSA-72766	Translation	125/1406	1.16E-29	7.16E-28	125	2	122
R-HSA-376176	Signaling by ROBO receptors	98/1406	1.99E-27	1.17E-25	98	6	91
R-HSA-5663205	Infectious disease	132/1406	1.65E-21	9.26E-20	132	15	116
R-HSA-8868773	rRNA processing in the nucleus and cytosol	80/1406	3.65E-18	1.96E-16	80	2	77
R-HSA-6791226	Major pathway of rRNA processing in the nucleolus and cytosol	77/1406	5.59E-18	2.88E-16	77	2	74
R-HSA-72312	rRNA processing	80/1406	1.27E-16	6.28E-15	80	2	77
R-HSA-71291	Metabolism of amino acids and derivatives	110/1406	1.16E-14	5.49E-13	110	5	104
R-HSA-72695	Formation of the ternary complex, and subsequently, the 43S complex	33/1406	1.64E-14	7.49E-13	33	0	32
R-HSA-72662	Activation of the mRNA upon binding of the cap-binding complex and e	35/1406	8.81E-14	3.89E-12	35	0	34
R-HSA-72649	Translation initiation complex formation	34/1406	3.30E-13	1.40E-11	34	0	33
R-HSA-163200	Respiratory electron transport, ATP synthesis by chemiosmotic couplin	52/1406	8.10E-13	3.33E-11	52	1	51
R-HSA-72702	Ribosomal scanning and start codon recognition	33/1406	2.30E-12	9.17E-11	33	0	32
R-HSA-6798695	Neutrophil degranulation	123/1406	1.68E-10	6.49E-09	123	30	93
R-HSA-611105	Respiratory electron transport	40/1406	3.45E-09	1.29E-07	40	1	39
R-HSA-1428517	The citric acid (TCA) cycle and respiratory electron transport	57/1406	6.30E-09	2.29E-07	57	2	55
R-HSA-162909	Host Interactions of HIV factors	42/1406	7.89E-07	2.78E-05	42	8	34
R-HSA-5628897	TP53 Regulates Metabolic Genes	30/1406	8.84E-06	0.000303	30	8	22
R-HSA-5368286	Mitochondrial translation initiation	30/1406	1.15E-05	0.000364	30	2	28
R-HSA-5389840	Mitochondrial translation elongation	30/1406	1.15E-05	0.000364	30	2	28
R-HSA-5419276	Mitochondrial translation termination	30/1406	1.15E-05	0.000364	30	2	28
R-HSA-163210	Formation of ATP by chemiosmotic coupling	11/1406	1.48E-05	0.000458	11	0	11
R-HSA-917937	Iron uptake and transport	21/1406	2.19E-05	0.000659	21	6	15
R-HSA-6799198	Complex I biogenesis	21/1406	4.23E-05	0.001242	21	0	21
R-HSA-5368287	Mitochondrial translation	30/1406	4.95E-05	0.001421	30	2	28
R-HSA-5627117	RHO GTPases Activate ROCKs	11/1406	5.77E-05	0.00162	11	4	7
R-HSA-5607764	CLEC7A (Dectin-1) signaling	31/1406	7.06E-05	0.001937	31	9	22
R-HSA-1592230	Mitochondrial biogenesis	30/1406	7.72E-05	0.002011	30	11	19
R-HSA-180585	Vif-mediated degradation of APOBEC3G	20/1406	7.78E-05	0.002011	20	1	19
R-HSA-1168372	Downstream signaling events of B Cell Receptor (BCR)	27/1406	7.82E-05	0.002011	27	7	20
R-HSA-8949613	Cristae formation	14/1406	9.67E-05	0.002437	14	0	14
R-HSA-416572	Sema4D induced cell migration and growth-cone collapse	11/1406	0.000104	0.00257	11	5	6
R-HSA-2871837	FCERI mediated NF-kB activation	26/1406	0.000134	0.003256	26	6	20
R-HSA-8941858	Regulation of RUNX3 expression and activity	20/1406	0.000142	0.003367	20	2	18
R-HSA-400685	Sema4D in semaphorin signaling	12/1406	0.000149	0.003428	12	5	7
R-HSA-5621481	C-type lectin receptors (CLRs)	38/1406	0.00015	0.003428	38	15	23
R-HSA-8878159	Transcriptional regulation by RUNX3	29/1406	0.000197	0.004413	29	9	20
R-HSA-8953897	Cellular responses to external stimuli	104/1406	0.00027	0.005961	104	39	65
R-HSA-166208	mTORC1-mediated signalling	11/1406	0.000294	0.006377	11	3	8
R-HSA-3299685	Detoxification of Reactive Oxygen Species	14/1406	0.000319	0.006627	14	3	11
R-HSA-2262752	Cellular responses to stress	90/1406	0.000321	0.006627	90	34	56
R-HSA-5688426	Deubiquitination	63/1406	0.000322	0.006627	63	34	29
R-HSA-450408	AUF1 (hnRNP D0) binds and destabilizes mRNA	19/1406	0.000442	0.008942	19	0	19
R-HSA-349425	Autodegradation of the E3 ubiquitin ligase COP1	18/1406	0.000461	0.009046	18	2	16
R-HSA-162906	HIV Infection	55/1406	0.000461	0.009046	55	12	43
R-HSA-69541	Stabilization of p53	19/1406	0.000572	0.011033	19	2	17
R-HSA-8854050	FBXL7 down-regulates AURKA during mitotic entry and in early mitosis	18/1406	0.000603	0.01134	18	0	18
R-HSA-1852241	Organelle biogenesis and maintenance	67/1406	0.000606	0.01134	67	30	37
R-HSA-165159	mTOR signalling	15/1406	0.000651	0.012002	15	6	9
[illegible]	[illegible]	[illegible]	[illegible]	[illegible]	[illegible]	[illegible]	[illegible]

Table S8 (continued).

ReactomeID	Description	GeneRatio	pvalue	padj	Count	Up	Down
R-HSA-446203	Asparagine N-linked glycosylation	66/1406	0.000687	0.012485	66	18	48
R-HSA-3858494	Beta-catenin independent WNT signaling	37/1406	0.000698	0.012497	37	5	32
R-HSA-983705	Signaling by the B Cell Receptor (BCR)	32/1406	0.000711	0.012545	32	10	22
R-HSA-1169091	Activation of NF-kappaB in B cells	21/1406	0.000792	0.01377	21	4	17
R-HSA-1236975	Antigen processing-Cross presentation	28/1406	0.000824	0.014139	28	4	24
R-HSA-373755	Semaphorin interactions	20/1406	0.000862	0.014349	20	8	12
R-HSA-5358346	Hedgehog ligand biogenesis	20/1406	0.000862	0.014349	20	2	18
R-HSA-209560	NF-kB is activated and signals survival	7/1406	0.000879	0.014349	7	3	4
R-HSA-6806003	Regulation of TP53 Expression and Degradation	14/1406	0.000883	0.014349	14	7	7
R-HSA-5357956	TNFR1-induced NFkappaB signaling pathway	12/1406	0.001157	0.018558	12	7	5
R-HSA-4086400	PCP/CE pathway	26/1406	0.001246	0.019724	26	1	25
R-HSA-5362768	Hh mutants that don't undergo autocatalytic processing are degraded b	18/1406	0.001276	0.019954	18	0	18
R-HSA-5689603	UCH proteinases	27/1406	0.001314	0.020177	27	7	20
R-HSA-69275	G2/M Transition	46/1406	0.001323	0.020177	46	16	30
R-HSA-9020702	Interleukin-1 signaling	28/1406	0.001373	0.020438	28	9	19
R-HSA-180534	Vpu mediated degradation of CD4	17/1406	0.001374	0.020438	17	0	17
R-HSA-175474	Assembly Of The HIV Virion	8/1406	0.001415	0.020806	8	1	7
R-HSA-4641258	Degradation of DVL	18/1406	0.00161	0.022827	18	0	18
R-HSA-5387390	Hh mutants abrogate ligand secretion	18/1406	0.00161	0.022827	18	0	18
R-HSA-1234174	Regulation of Hypoxia-inducible Factor (HIF) by oxygen	22/1406	0.001641	0.022827	22	2	20
R-HSA-2262749	Cellular response to hypoxia	22/1406	0.001641	0.022827	22	2	20
R-HSA-1234176	Oxygen-dependent proline hydroxylation of Hypoxia-inducible Factor A	20/1406	0.00166	0.022827	20	0	20
R-HSA-453274	Mitotic G2-G2/M phases	46/1406	0.001664	0.022827	46	16	30
R-HSA-69601	Ubiquitin Mediated Degradation of Phosphorylated Cdc25A	17/1406	0.001748	0.02321	17	1	16
R-HSA-69610	p53-Independent DNA Damage Response	17/1406	0.001748	0.02321	17	1	16
R-HSA-69613	p53-Independent G1/S DNA damage checkpoint	17/1406	0.001748	0.02321	17	1	16
R-HSA-5625900	RHO GTPases activate CIT	9/1406	0.001814	0.023802	9	3	6
R-HSA-8939902	Regulation of RUNX2 expression and activity	21/1406	0.001831	0.023802	21	3	18
R-HSA-381340	Transcriptional regulation of white adipocyte differentiation	24/1406	0.001886	0.024259	24	15	9
R-HSA-202424	Downstream TCR signaling	26/1406	0.002075	0.026423	26	5	21
R-HSA-5689880	Ub-specific processing proteases	45/1406	0.002118	0.026696	45	22	23
R-HSA-6804757	Regulation of TP53 Degradation	13/1406	0.002197	0.027408	13	6	7
R-HSA-5625740	RHO GTPases activate PKNs	24/1406	0.002245	0.027731	24	10	14
R-HSA-211733	Regulation of activated PAK-2p34 by proteasome mediated degradatior	16/1406	0.002396	0.029147	16	0	16
R-HSA-195258	RHO GTPase Effectors	66/1406	0.002476	0.029147	66	28	38
R-HSA-5610780	Degradation of GLI1 by the proteasome	18/1406	0.002502	0.029147	18	0	18
R-HSA-5610783	Degradation of GLI2 by the proteasome	18/1406	0.002502	0.029147	18	0	18
R-HSA-5610785	GLI3 is processed to GLI3R by the proteasome	18/1406	0.002502	0.029147	18	0	18
R-HSA-5676590	NIK-->noncanonical NF-kB signaling	18/1406	0.002502	0.029147	18	1	17
R-HSA-195253	Degradation of beta-catenin by the destruction complex	23/1406	0.002535	0.029264	23	3	20
R-HSA-174113	SCF-beta-TrCP mediated degradation of Emi1	17/1406	0.002756	0.031225	17	0	17
R-HSA-4641257	Degradation of AXIN	17/1406	0.002756	0.031225	17	1	16
R-HSA-5357905	Regulation of TNFR1 signaling	12/1406	0.003012	0.033399	12	7	5
R-HSA-8948751	Regulation of PTEN stability and activity	20/1406	0.003013	0.033399	20	3	17
R-HSA-380972	Energy dependent regulation of mTOR by LKB1-AMPK	11/1406	0.003043	0.033399	11	4	7
R-HSA-5607761	Dectin-1 mediated noncanonical NF-kB signaling	18/1406	0.003083	0.033399	18	1	17
R-HSA-5678895	Defective CFTR causes cystic fibrosis	18/1406	0.003083	0.033399	18	0	18
R-HSA-6807070	PTEN Regulation	34/1406	0.003499	0.037574	34	9	25
R-HSA-187577	SCF(Skp2)-mediated degradation of p27/p21	18/1406	0.003772	0.039493	18	0	18
R-HSA-69229	Ubiquitin-dependent degradation of Cyclin D1	16/1406	0.003773	0.039493	16	0	16
R-HSA-75815	Ubiquitin-dependent degradation of Cyclin D	16/1406	0.003773	0.039493	16	0	16
R-HSA-8852276	The role of GTSE1 in G2/M progression after G2 checkpoint	21/1406	0.003852	0.039977	21	1	20
R-HSA-1368082	RORA activates gene expression	9/1406	0.003996	0.040509	9	9	0
R-HSA-432720	Lysosome Vesicle Biogenesis	12/1406	0.004002	0.040509	12	1	11
R-HSA-936440	Negative regulators of DDX58/IFIH1 signaling	12/1406	0.004002	0.040509	12	6	6
R-HSA-69563	p53-Dependent G1 DNA Damage Response	19/1406	0.004083	0.040666	19	2	17
R-HSA-69580	p53-Dependent G1/S DNA damage checkpoint	19/1406	0.004083	0.040666	19	2	17
R-HSA-8866652	Synthesis of active ubiquitin: roles of E1 and E2 enzymes	11/1406	0.004134	0.040845	11	2	9
R-HSA-400253	Circadian Clock	20/1406	0.004351	0.042521	20	13	7
R-HSA-195721	Signaling by WNT	64/1406	0.004373	0.042521	64	21	43
R-HSA-1989781	PPARA activates gene expression	28/1406	0.004584	0.044229	28	20	8
R-HSA-193639	p75NTR signals via NF-kB	7/1406	0.004629	0.044319	7	3	4
R-HSA-169911	Regulation of Apoptosis	16/1406	0.004674	0.044401	16	0	16
R-HSA-174154	APC/C:Cdc20 mediated degradation of Securin	19/1406	0.00491	0.046288	19	0	19
R-HSA-5668541	TNFR2 non-canonical NF-kB pathway	25/1406	0.00513	0.047435	25	8	17
R-HSA-174178	APC/C:Cdh1 mediated degradation of Cdc20 and other APC/C:Cdh1 tar	20/1406	0.005185	0.047435	20	0	20
R-HSA-199992	trans-Golgi Network Vesicle Budding	20/1406	0.005185	0.047435	20	2	18
R-HSA-421837	Clathrin derived vesicle budding	20/1406	0.005185	0.047435	20	2	18

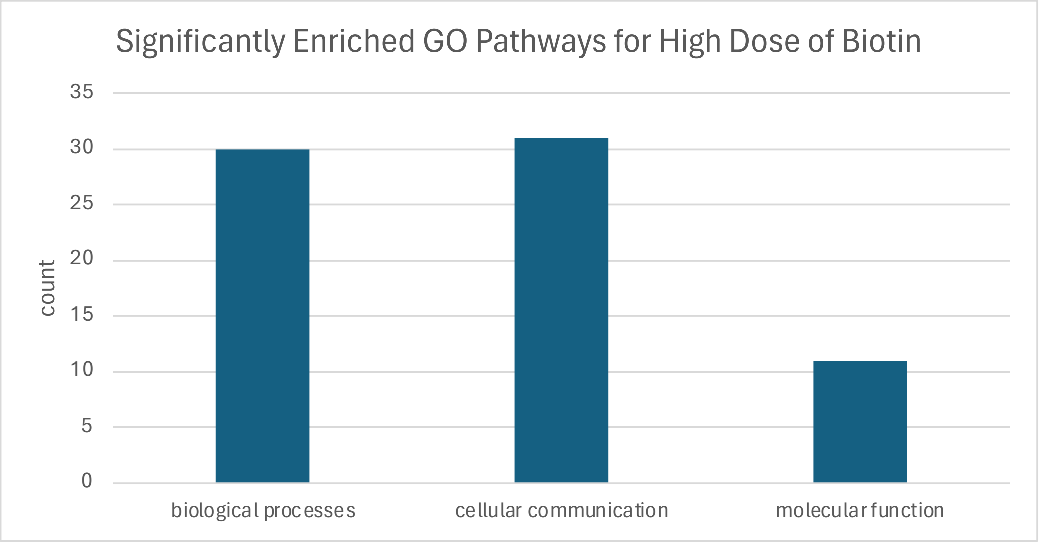

Figure S4: Number of significantly enriched gene ontology pathways in each classification for the treatment of macrophages with a high dose of biotin. GO, gene ontology.

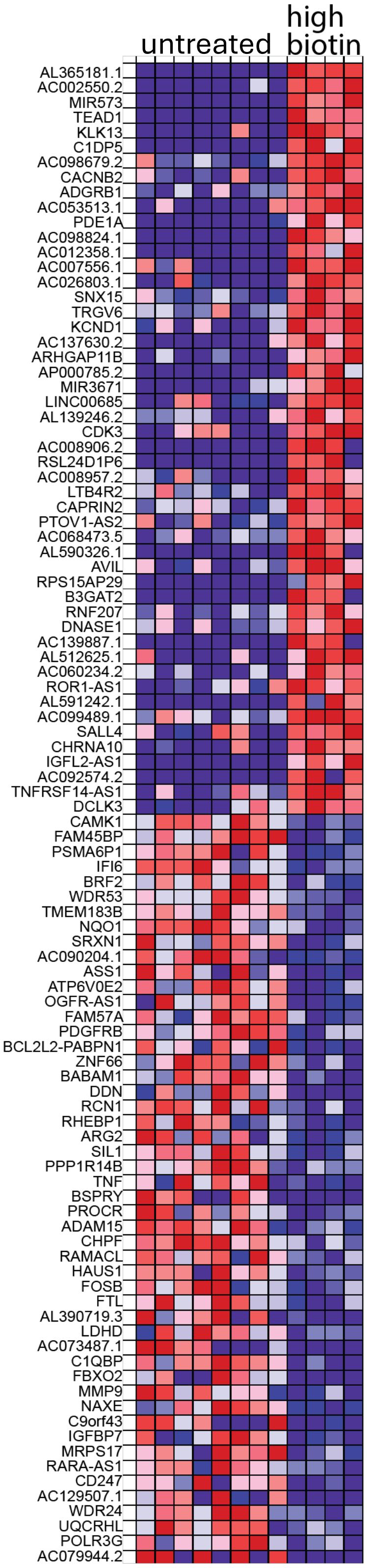

Figure S5: Heatmap of most significantly enriched genes, determined by gene set enrichment analysis (GSEA), for top 50 most significantly enriched GO pathways for high biotin treatment for each donor **(**n=8, 4 male and 4 female).

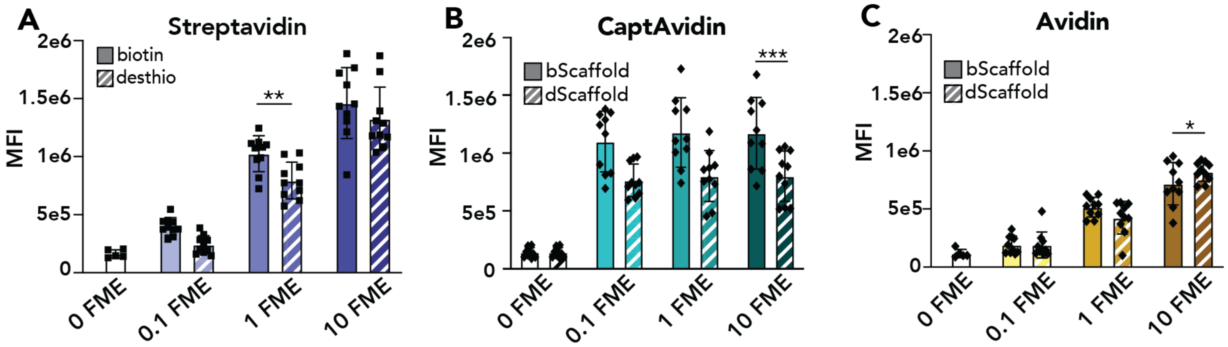

Figure S6: Fluorescent avidin variant bound to modified porous gelatin scaffolds. (**A - F**) Fluorescent intensity of avidin, streptavidin, or CaptAvidin bound to biotinylated or desthiobiotinylated scaffolds with increasing biotin densities. Data represented as mean +/- SD with n = 10. Statistical significance determined using Ordinary two-way ANOVA followed by Tukey's multiple comparison test. *p<0.05, **p<0.01, ***p<0.001, ****p<0.0001. bScaffold, biotinylated scaffold. dScaffold, desthiobiotinylated scaffold. MFI, mean fluorescent intensity. FME, fold molar excess. strep, streptavidin. Capt, CaptAvidin.

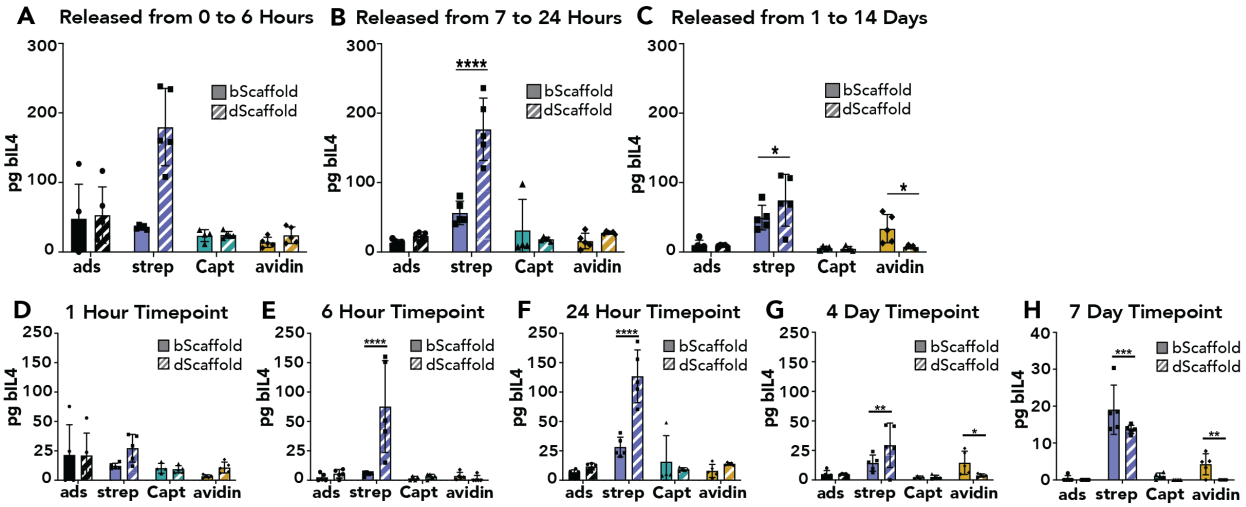

Figure S7: Release of biotinylated IL-4 from modified scaffolds. (**A-C**) Release of biotinylated IL-4 (bIL-4) from either biotinylated or desthiobiotinylated scaffolds bound with avidin, streptavidin, or CaptAvidin with adsorbed bIL-4 acting as a control. (**D-I**) Mass of bIL-4 released from either biotinylated or desthiobiotinylated scaffolds bound with avidin, streptavidin, or CaptAvidin with adsorbed bIL-4 acting as a control at each timepoint over 14 days. Data represented as mean +/- SD with n = 5. Statistical significance determined using Ordinary two-way ANOVA followed by Tukey's multiple comparisons test. *p<0.05, **p<0.01, ***p<0.001, ****p<0.0001. bScaffold, biotinylated scaffold. dScaffold, desthiobiotinylated scaffold. ads, adsorbed. strep, streptavidin. Capt, CaptAvidin.

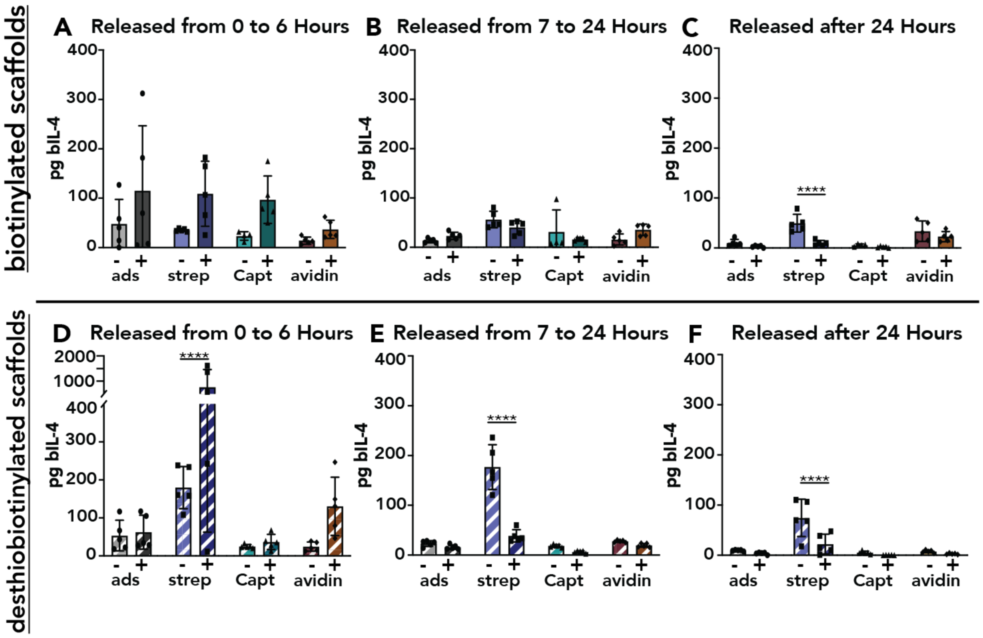

Figure S8: Release of biotinylated IL-4 from modified scaffolds in the presence of free biotin. (**A-F**) Release of biotinylated IL-4 (bIL-4) from either biotinylated or desthiobiotinylated scaffolds bound with avidin, streptavidin, or CaptAvidin with adsorbed bIL-4 acting as a control at each timepoint over 14 days. Data represented as mean +/- SD with n = 5. Statistical significance determined using Ordinary two-way ANOVA followed by Tukey's multiple comparisons test. ****p<0.0001.

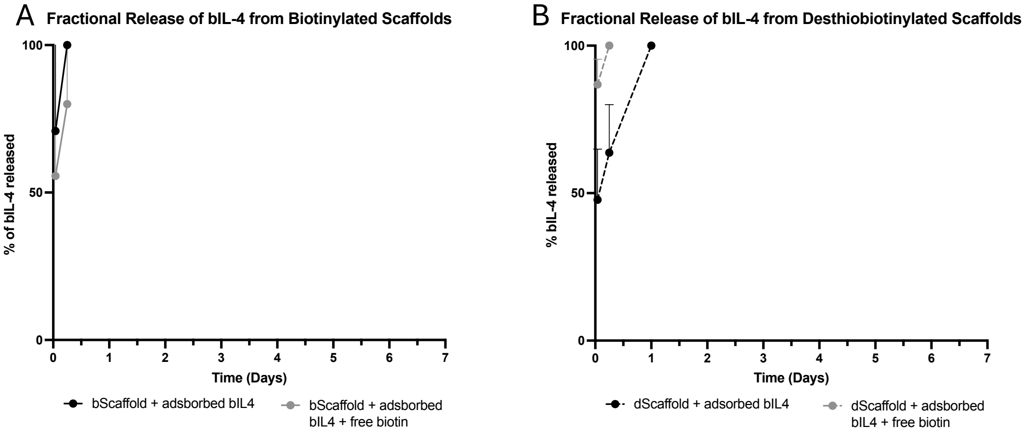

Figure S9: Fractional release curves of biotinylated IL-4 (bIL-4) from biotinylated or desthiobiotinylated scaffolds in the absence or presence of 10mM free biotin. (**A**) Desorption of bIL-4 from biotinylated scaffolds in the absence or presence of 10mM free biotin in solution (**B**) Desorption of bIL-4 from biotinylated scaffolds in the absence or presence of 10mM free biotin in solution. bScaffold, biotinylated scaffold. dScaffold, desthiobiotinylated scaffold.

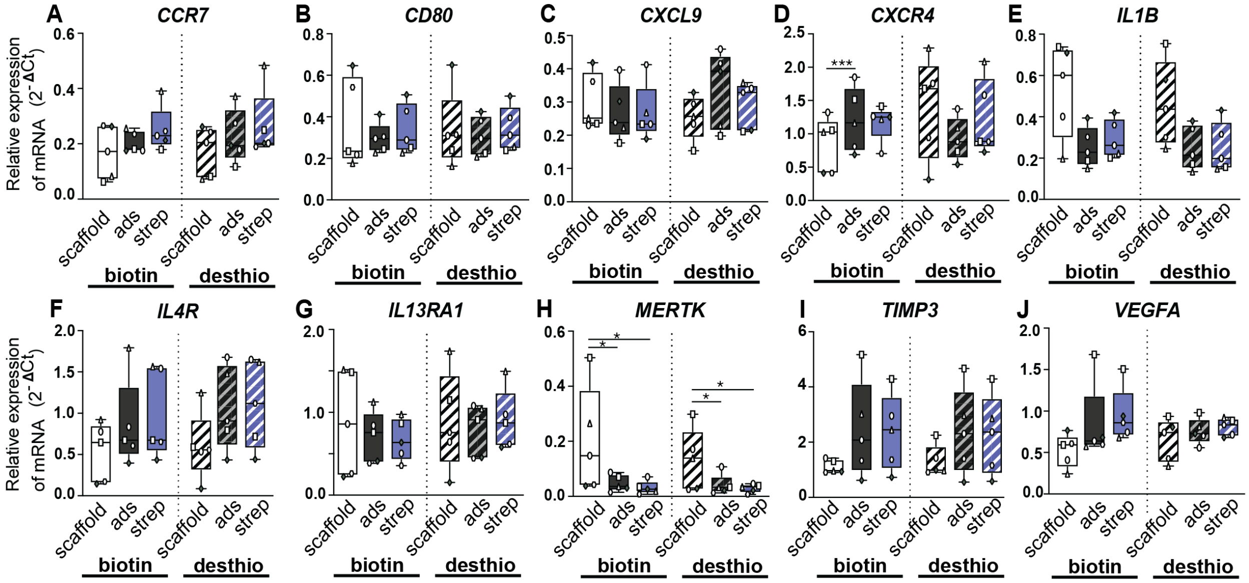

Figure S10: Expression of non-IL-4 driven genes by qRT-PCR. (**A** - **J**) Relative expression of mRNA from M0 macrophages seeded onto either a scaffold (biotinylated or desthiobiotinylated scaffold), adsorbed bIL-4 (biotinylated or desthiobiotinylated scaffold with adsorbed bIL-4), or streptavidin-bIL-4 (biotinylated or desthiobiotinylated scaffold) modified scaffold. Data represented as mean +/- SD with n = 5. 4 male (white) and 1 female (blue) with each donor represented by a unique symbol. Statistical significance determined using RM two-way ANOVA with the Geisser-Greenhouse correction, followed by Tukey's multiple comparison test. *p<0.05, **p<0.01, ***p<0.001, ****p<0.0001. scaffold, indicates either a biotinylated or desthiobiotinylated scaffold. biotin, biotinylated scaffold. desthio, desthiobiotinylated scaffold. ads, adsorbed bIL-4. strep, streptavidin.

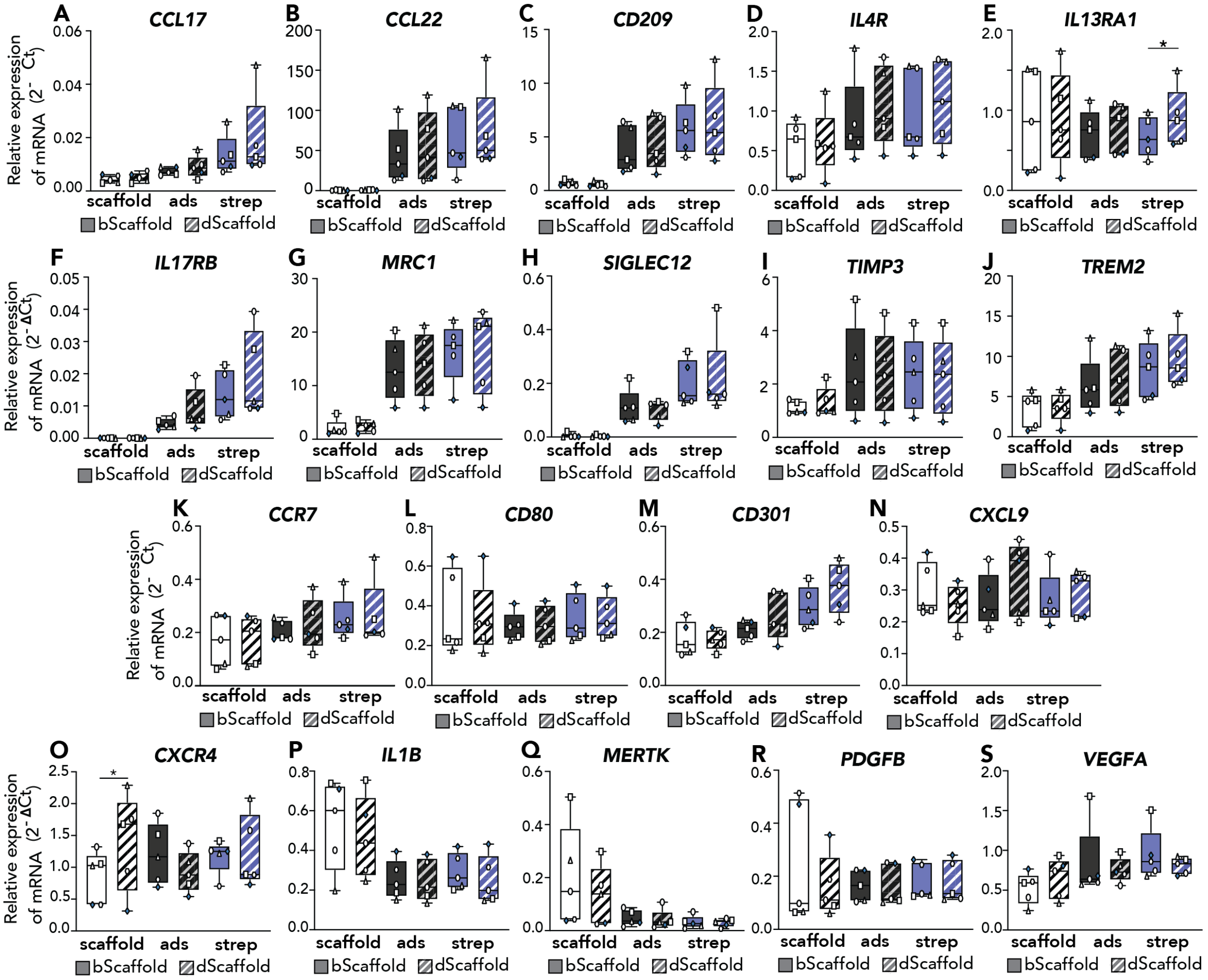

Figure S11: Gene expression from macrophages seeded onto modified scaffolds for 4 days by qRT-PCR. (**A - S**) Relative expression of genes from macrophages seeded onto either a control (biotinylated or desthiobiotinylated scaffold), adsorbed bIL-4 (biotinylated or desthiobiotinylated scaffold with adsorbed bIL-4), or streptavidin-bIL-4 (biotinylated or desthiobiotinylated scaffold) modified scaffold. Data represented as mean +/- SD with n = 5, 4 male (white) and 1 female (blue) with each donor represented by a unique symbol. Statistical significance determined using RM two-way ANOVA with the Geisser-Greenhouse correction, followed by Tukey's multiple comparison test. *p<0.05, **p<0.01, ***p<0.001, ****p<0.0001. bScaffold, biotinylated scaffold. dScaffold, desthiobiotinylated scaffold. +bIL-4, adsorbead bIL-4. +strep +bIL-4, bound streptavidin with bIL-4.
